# Dysregulation of the dopaminergic system secondary to traumatic brain injury: implications for mood and anxiety disorders

**DOI:** 10.3389/fnins.2024.1447688

**Published:** 2024-08-08

**Authors:** Alfonso Mata-Bermudez, Ricardo Trejo-Chávez, Marina Martínez-Vargas, Adán Pérez-Arredondo, Maria de Los Ángeles Martínez-Cardenas, Araceli Diaz-Ruiz, Camilo Rios, Luz Navarro

**Affiliations:** ^1^Departamento de Fisiología Facultad de Medicina, Universidad Nacional Autónoma de México, Ciudad de México, Mexico; ^2^Doctorado en Ciencias Biomedicas, Universidad Nacional Autónoma de México, Ciudad de México, Mexico; ^3^Departamento de Atención a la Salud, Universidad Autónoma Metropolitana Unidad Xochimilco, Ciudad de México, Mexico; ^4^Departamento de Neuroquímica, Instituto Nacional de Neurología y Neurocirugía Manuel Velasco Suarez, Ciudad de México, Mexico; ^5^Laboratorio de Neurofarmacología Molecular, Departamento de Sistemas Biológicos, Universidad Autónoma Metropolitana Unidad Xochimilco, Ciudad de México, Mexico; ^6^Dirección de Investigación, Instituto Nacional de Rehabilitación Luis Guillermo Ibarra, Ciudad de México, Mexico

**Keywords:** traumatic brain injury, depression, mood disorders, anxiety, dopamine

## Abstract

Traumatic brain injury (TBI) represents a public health issue with a high mortality rate and severe neurological and psychiatric consequences. Mood and anxiety disorders are some of the most frequently reported. Primary and secondary damage can cause a loss of neurons and glial cells, leading to dysfunction of neuronal circuits, which can induce imbalances in many neurotransmitter systems. Monoaminergic systems, especially the dopaminergic system, are some of the most involved in the pathogenesis of neuropsychiatric and cognitive symptoms after TBI. In this work, we summarize the studies carried out in patients who have suffered TBI and describe alterations in the dopaminergic system, highlighting (1) dysfunction of the dopaminergic neuronal circuits caused by TBI, where modifications are shown in the dopamine transporter (DAT) and alterations in the expression of dopamine receptor 2 (D2R) in brain areas with dopaminergic innervation, thus establishing a hypodopaminergic state and (2) variations in the concentration of dopamine and its metabolites in biological fluids of post-TBI patients, such as elevated dopamine (DA) and alterations in homovanillic acid (HVA). On the other hand, we show a large number of reports of alterations in the dopaminergic system after a TBI in animal models, in which modifications in the levels of DA, DAT, and HVA have been reported, as well as alterations in the expression of tyrosine hydroxylase (TH). We also describe the biological pathways, neuronal circuits, and molecular mechanisms potentially involved in mood and anxiety disorders that occur after TBI and are associated with alterations of the dopaminergic system in clinical studies and animal models. We describe the changes that occur in the clinical picture of post-TBI patients, such as alterations in mood and anxiety associated with DAT activity in the striatum, the relationship between post-TBI major depressive disorders (MDD) with lower availability of the DA receptors D2R and D3R in the caudate and thalamus, as well as a decrease in the volume of the substantia nigra (SN) associated with anxiety symptoms. With these findings, we discuss the possible relationship between the disorders caused by alterations in the dopaminergic system in patients with TBI.

## 1 Introduction

Traumatic brain injury (TBI) is a significant public health problem, affecting millions of people around the world each year. The consequences of a traumatic brain injury extend beyond the immediate physical damage and cause long-term cognitive, emotional, and behavioral complications. Among them, dysregulation of the dopaminergic system has attracted significant attention due to its profound impact on neuropsychiatric outcomes (Bales et al., 2009; Corrigan et al., 2010). Dopamine (DA), a key neurotransmitter, regulates mood, motivation, reward processing, and executive functions. Alterations in dopaminergic signaling are increasingly recognized as key contributors to the pathophysiology of mood and anxiety disorders, conditions frequently observed in TBI survivors (Schultz, 1998; Nieoullon, 2002).

The dopaminergic system includes several critical pathways, including mesolimbic, mesocortical, and nigrostriatal, integral to various neurological and psychological processes. The mesolimbic pathway, often associated with the brain’s reward system, is crucial to motivation and the experience of pleasure. In contrast, the mesocortical pathway is vital for executive functions and cognitive control, while the nigrostriatal pathway predominantly influences motor control but also affects cognitive and emotional responses (Grace and Rosenkranz, 2002; Dunlop and Nemeroff, 2007). Alterations in these pathways after TBI can lead to significant neuropsychiatric sequelae, including depression and anxiety disorders.

Depression and anxiety disorders are particularly prevalent among post-TBI patients, and several studies indicate high rates of comorbidity that complicate recovery and rehabilitation efforts (Green et al., 2008; Ruttan et al., 2008). The intricate interplay between TBI-induced structural brain changes and subsequent dopaminergic dysregulation underscores the complexity of treating these conditions. Furthermore, the chronic nature of these psychiatric disorders following traumatic brain injury highlights the need for a greater understanding of the underlying neuropathological mechanisms (Fleminger and Ponsford, 2005).

Relatively recent and excellent reviews on disorders associated with the deregulation of the dopaminergic system secondary to traumatic brain injury focus on cognitive disorders (Bales et al., 2009, 2010; Chen et al., 2017b; Lan et al., 2019; Verduzco-Mendoza et al., 2021). However, mood and anxiety disorders after traumatic brain injury have not been described thoroughly. So, in this narrative review, we aim to provide a comprehensive overview of the current understanding of dopaminergic dysregulation following TBI and its implications for mood and anxiety disorders. We include the most recent clinical studies and original studies in rodents on the alterations described in the dopaminergic system secondary to TBI, and we highlight those in which these may be related to mood and anxiety disorders secondary to TBI. We also include a brief description of the pathophysiology of TBI, mood, and anxiety disorders and a succinct review of the dopaminergic system.

## 2 Traumatic brain injury

TBI is defined as a disruption of brain function or any other pathological evidence that emerges from an external physical force that may result in temporary or permanent impairment (Khellaf et al., 2019; Capizzi et al., 2020). This condition represents a public health issue with a high mortality rate and severe neurological and psychiatric consequences in the short and long term, drastically reducing the quality of life of patients (Osborn et al., 2014; Perry et al., 2016; Dixon, 2017). The estimated annual incidence of TBI worldwide is 69 million people, representing 939 cases per 100,000, of which 55.9 million correspond to mild TBI and 5.48 million to severe TBI (Dewan et al., 2018). Likewise, it has been reported that the most affected populations correspond to young men between 15 and 25 years old, followed by older adults and infants. The leading causes of this issue are car accidents, falls, violence, and sports and recreational accidents (Azouvi et al., 2017).

TBI originates when the head is subjected to an external mechanical force that can cause any degree of injury to brain tissue (Agrawal and Branco, 2016). The primary injury determines the morbidity and mortality of TBI, originating at the time of trauma, or the secondary injury, whose effect appears later (Leker and Shohami, 2002). Primary injury, which occurs at the time of impact and is not reversible, includes tearing of white matter, cortical contusion, axonal damage, intracerebral, epidural, and subdural hematoma, subarachnoid hemorrhage, intraventricular hemorrhage, and diffuse edema. At the cellular level, early events of neurotrauma include microporation of membranes, misalignment of ion channels, and protein conformational changes (Maas et al., 2008). With further damage, blood vessels can tear, causing microbleeds and ischemia of brain tissue, which can be extensive or, more commonly, perilesional (Menon et al., 2010; Pinto et al., 2012; Agrawal and Branco, 2016).

Unlike primary injury, secondary injury, which corresponds to late effects (minutes to months), is a potentially reversible process through adequate therapy (Leker and Shohami, 2002). Secondary brain injury induces glial inflammatory responses as well as necrosis and apoptosis of neurons and glial cells, such as oligodendrocytes, leading to demyelination and loss or dysfunction of neuronal circuits (Pavlovic et al., 2019); this could induce an imbalance of monoaminergic systems involved in the pathogenesis of neuropsychiatric and cognitive symptoms after a TBI (Jenkins et al., 2016). In addition, brain injuries can progress for weeks or months after the initial damage extends to vulnerable brain regions, such as the prefrontal cortex, hippocampus, thalamus, striatum, amygdala, and forebrain nuclei involved in the subject’s cognitive and emotional functioning.

## 3 Psychiatric manifestations secondary to TBI

There is extensive literature about the cognitive change observed after TBI, including deficits in executive functions, attention, and working memory (Cristofori and Levin, 2015). However, changes in emotional processing after TBI have been less well described. Although they may result in poorly integrated self-representation and dysfunctional interpersonal relationships, increasing the patient’s vulnerability to developing affective disorders (Jorge, 2015). Acquired neurological injuries secondary to TBI are associated with a broad spectrum of pathologies in mood and other psychiatric manifestations (Donnemiller et al., 2000). Depression and anxiety disorders occupy first and second place among the most common neuropsychiatric disorders in post-TBI patients (Mallya et al., 2015; Braga et al., 2022).

Among TBI’s most common and difficult-to-treat long-term psychiatric complications are depressive disorders, with an estimated prevalence of 20 to 45% (Kreitzer et al., 2019). Anxiety disorders are more common in patients who have suffered a mild TBI than a severe one, in contrast to cognitive impairments that increase with the severity of the injury (Bryant et al., 2010; Corrigan et al., 2023). Although the mechanisms underlying the association of TBI and depressive behaviors are still unclear (Jahan and Tanev, 2023), there is evidence in various animal models that depression is associated with a functional deficit of monoamines (noradrenaline, NA; serotonin, 5-HT, and DA) in specific brain sites (Traeger et al., 2020).

Regarding DA, Bales et al. (2009, 2010) proposed a hypothesis where changes in the neurotransmission of this molecule are reflected in the development of cognitive and behavioral dysfunction and emotional lability in subjects who suffered a TBI. Sequelae observed may depend on the extent of the injury on the dopaminergic pathway(s) involved. Alterations of the nigrostriatal pathway include deficits in working memory and emotional and behavioral issues, usually correlated with low concentrations of DA; manifestations observed as dysfunction of the mesolimbic and mesocortical pathways are depression, anxiety, and substance abuse, associated with loss of DA homeostasis (Chen et al., 2017b, 2018). Some of the behavioral or personality disorders secondary to TBI are attributed to executive dysfunction associated with diffuse axonal damage and interruption of corticostriatal connectivity (Pischiutta et al., 2018), but the neural bases of these problems and others have remained uncertain; similarly, changes in mood, anxiety or depression associated with TBI can be the outcome of events as prominent as diffuse axonal damage or interruption of neuronal circuits within dopaminergic pathways (Chen et al., 2017b).

These data underpin the importance of dopaminergic transmission in post-TBI disorders. However, it is worth noting that there potentially exists a significant heterogeneity of mechanisms leading to depression/anxiety following TBI (Jahan and Tanev, 2023).

Likewise, another factor to consider is the patient’s neuropsychiatric state before the TBI. It has been described that the rate of neuropsychiatric disorders is much higher among patients with TBI than among subjects without TBI, even before the TBI occurs (Albrecht et al., 2020). Besides, it is worth noting that injuries occurring in a psychologically traumatic context could independently confer risk for mood disorders. In a recent study of patients with mild TBI or with orthopedic injuries not involving the head, it was reported that patients with mild TBI were more likely to report Posttraumatic Stress Disorder (PTSD) and/or major depressive symptoms, especially those with prior mental health problems, or when the TBI was associated with an assault or other violent cause (Stein et al., 2019).

## 4 Mood or affective and anxiety disorders

The mood has been defined as the internal and sustained emotional state or tone of a person that gives “color” to their perception of being part of the world, whereas the affective state is the expression of the mood, being interpreted by the clinician as the patient’s state of mind. Some authors have suggested combining both elements in a new name: “emotional expression,” and many others use them interchangeably (Sadock et al., 2015). Anxiety is an anticipatory response to a future threat; it differs from fear in responding to an imminent threat.

Mood or affective disorders are described as overt disturbances in emotions (severe dips called depression or ups and downs called hypomania or mania). According to the Diagnostic and Statistical Manual of Mental Disorders, Fifth Edition (DSM-5), mood disorders are categorized into (1) bipolar and related disorders and (2) depressive disorders (Spijker and Claes, 2014; Sekhon and Gupta, 2023). Even though anxiety is a common symptom in some depressive disorders, anxiety disorders are considered a family of mental disorders different from affective disorders, according to the DSM-5.

## 5 Mood and anxiety disorders associated with traumatic brain injury

For many TBI survivors, sensorimotor and neurological consequences developed over time can lead to mood and anxiety imbalances that severely affect their quality of life. These behavioral changes vary depending on the type of injury and the type of injured brain tissue. Protracted recovery after a traumatic brain event has been consistently linked with the emotional capacity of individuals to cope with possible physical (mobility), psychiatric, and social impairments (Yehene et al., 2020). Also, the ability to accept a possible disability prevents the normal reintegration of patients into society (Yehene et al., 2020).

Depressive disorders associated with TBI are generally classified, according to the DSM-5, as mood disorders due to another medical condition, with subtypes: (1) major depression, (2) prominent depressed mood, and (3) mixed features. TBI-related bipolar disorders are subdivided into (1) manic or hypomanic episodes, (2) manic features, or (3) mixed characteristics (Jorge, 2015). At the same time, anxiety disorders fall under the “due to another medical condition” classification.

Various studies report that during the months after TBI, anxiety and post-traumatic stress frequently occur and can persist for several years, significantly deteriorating the quality of life of patients and the people around them (Whelan-Goodinson et al., 2009; Huang et al., 2020; Robert, 2020).

Depression is a mood disorder that causes persistent sadness, constant negative thoughts, apathy, lack of energy, cognitive distortions, nihilism, and inability to enjoy everyday life events (Guillamondegui et al., 2011). In patients with TBI, depression is one of the most common disorders. Its treatment can be challenging since its etiology is uncertain and multifactorial and can develop through multiple pathways that are not necessarily the product of the primary brain lesion (Juengst et al., 2017). The risk of developing depressive disorders in adults who have suffered moderate to severe TBI has been reported to range from 66 to 73% in the first 6 months after the trauma (Abdullah et al., 2018; Ryttersgaard et al., 2020; Eliasen et al., 2021; Sameh et al., 2021). In 2016, Perry reviewed 57 studies and reported an odds ratio of 2.14 for the relationship between depression and having suffered a TBI (Perry et al., 2016). Some studies have focused on a specific age group; namely, in the elderly (≥65 years), the risk ranges between 16 and 29% (Vadlamani and Albrecht, 2020), while in the pediatric population, 33 to 50% (Laliberté Durish et al., 2018). Although anxiety is the most common affective disorder diagnosed within the first 12 months after TBI (Lamontagne et al., 2022), its onset can arise several years later (Albicini and McKinlay, 2018). The incidence of this condition ranges between 11 and 17% in adults after the occurrence of a TBI (Osborn et al., 2016; Albrecht et al., 2017; Abdullah et al., 2018; Ponsford et al., 2020), whereas the incidence in children and adolescents between 5 and 18 years old is 8% (Albicini et al., 2020).

Despite the devastating and disabling consequences of these disorders, their follow-up is often not adequate in clinical practice, especially for those patients who have suffered a mild TBI. Mood and anxiety disorders resulting from brain injury are multifactorial. Therefore, to propose pharmacological treatment, it is necessary to identify the involved neurobiological systems succeeding TBI (Juengst et al., 2017). For example, structural and functional changes that occur after TBI due to the initial mechanical damage induce the interruption of neural circuits, mainly in the prefrontal cortex (Koenigs and Grafman, 2009), the hippocampus (Hercher et al., 2009), the striatum (Carlson and Dixon, 2018; Leconte et al., 2020), and the thalamus (Osborn et al., 2014). These brain nuclei are closely related to dopaminergic neurotransmission (Hasler et al., 2008).

## 6 Dopaminergic system

DA is one of the most important neurotransmitters in the central nervous system (CNS), and its dysregulation has been consistently associated with the development of diverse neurological and psychiatric diseases (Klein et al., 2019).

DA is synthesized in the cytosol of dopaminergic neurons when tyrosine hydroxylase (TH) catalyzes the addition of a hydroxyl group at the meta position of tyrosine to produce L-3,4-dihydroxyphenylalanine (L-DOPA). Subsequently, L-DOPA is decarboxylated by the enzyme DOPA decarboxylase (DDC), synthesizing DA (Harsing, 2008; Meiser et al., 2013). Once DA is synthesized in the neuronal cytoplasm, it is stored in synaptic vesicles within presynaptic terminals by vesicular monoamine transporter-2 (VMAT2), located in the membrane of cell vesicles where it is stored and protected from intraneuronal deamination (Mulvihill, 2019). Once released, DA excites dopaminergic neurons through interactions with postsynaptic receptors and undergoes a reuptake process to stop signaling via the DA transporter (DAT; Mulvihill, 2019). DAT is a transmembrane protein expressed exclusively in dopaminergic neurons (Harsing, 2008). The remaining DA still in the cytosol is degraded by the action of monoamine oxidase (MAO) through an oxidative deamination process (Meiser et al., 2013). Also, DA can be taken up by surrounding glial cells. Glia readily degrades DA by the enzymatic action of MAO and by catechol-O-methyl transferase (COMT). The primary metabolites derived from DA degradation are dihydroxyphenylacetic acid (DOPAC) and homovanillic acid (HVA; Myöhänen et al., 2010).

### 6.1 Dopaminergic receptors

Five types of dopaminergic receptors (D1R, D2R, D3R, D4R, and D5R) belonging to the family of G protein-coupled receptors have been identified, located on dendrites, cell bodies of neurons, and axons or nerve terminals. These receptors are divided according to differences in their characteristics and signaling mechanisms. The family of D1-like receptors includes subtypes D1R and D5R, being associated with the increase of cyclic adenosine monophosphate (cAMP) through Gs proteins, stimulating the activation of adenylate cyclase (AC) in the striatal and retinal membranes. D1R is closely related to motor function, growth, development, reward, sleep, impulse control, reproductive behavior, working memory, and learning (Rodrigues et al., 2007; Ortiz et al., 2010; Ayano, 2016). The highest density of D1R is found in the mesolimbic, nigrostriatal, and mesocortical areas, which include the substantia nigra (SN), olfactory bulb, nucleus accumbens (NAc), caudate, putamen, and nucleus striatum (Ayano, 2016).

Conversely, D5R is expressed mainly in the SN, hypothalamus, hippocampus, and sympathetic ganglia. Various data indicate that these receptors are involved in nociceptive, affective, and endocrine processes regulated by DA (Ayano, 2016).

The family of D2-like receptors is formed by dopaminergic receptors D2R, D3R, and D4R; these are coupled to a Gi protein, which inhibits AC. D2R modulates working memory, reward-motivational functions, renal functions, gastrointestinal motility, vasodilation, and locomotion. These receptors have a higher expression in the SN, the olfactory bulb, the caudate, the putamen, the ventral tegmental area (VTA), and NAc (Ayano, 2016). In comparison, D3R is involved in endocrine function, emotions, and regulation of motor functions. They are expressed mainly in the islands of Calleja, the septal region, the medial and lateral geniculate nuclei of the thalamus, the medial mammillary nucleus of the hypothalamus, and Purkinje cells of the cerebellum (Ayano, 2016). Finally, D4R is related to cognitive and emotional functions and the reward system. They are expressed mainly in the SN, the hippocampus, the amygdala, the thalamus, the hypothalamus, the cerebral cortex, the sympathetic ganglia, and the globus pallidus (GP; Harsing, 2008).

### 6.2 Dopaminergic neurotransmission

**Figure 1:**
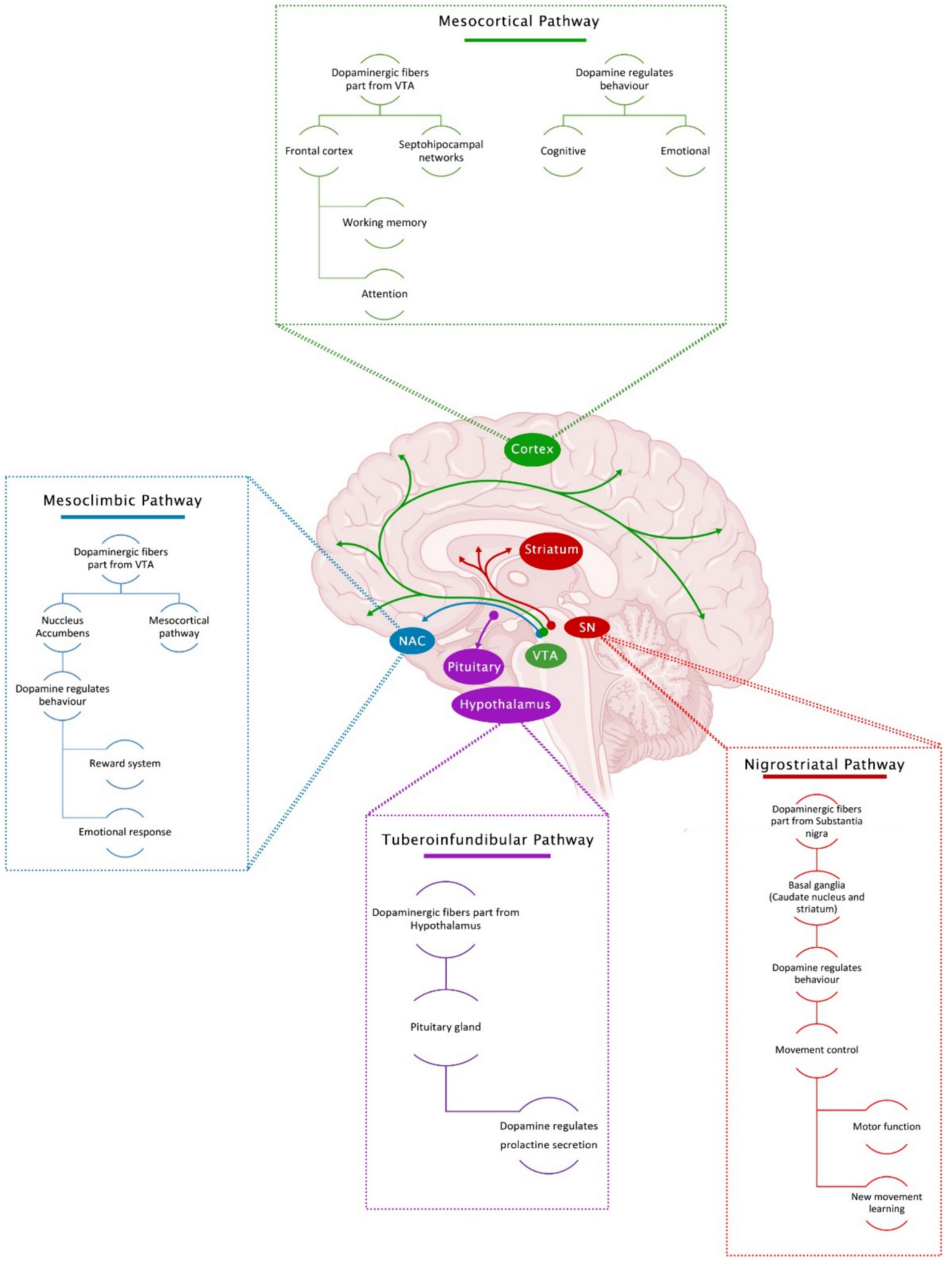
Main dopaminergic pathways in the brain and associated functions.

Dopaminergic innervation is the most prominent in the brain. Four main dopaminergic pathways have been identified (Figure 1).

#### 6.2.1 Nigrostriatal pathway

Here, dopaminergic projections are originated in the substantia nigra pars compacta (SNc) and are projected rostrally to be widely distributed in the basal ganglia (caudate nucleus and putamen) (Ko and Strafella, 2012). In this pathway, DA cell bodies send ascending projections to the striatum, playing an essential role in movement, especially in motor function control and learning new movement skills (Ayano, 2016).

#### 6.2.2 Mesolimbic pathway

Within the mesolimbic pathway, dopaminergic projections originate in the mesencephalon’s VTA. They are projected towards the mesocortical and limbic system (through the NAc) for their subsequent dissemination in the amygdala, the piriform cortex, the septal nuclei sides, and the NAc. These neurons are closely involved in several primary CNS functions, including voluntary movement, feeding, affection, reward, sleep, attention, working memory, and learning (Beaulieu and Gainetdinov, 2011). This pathway regulates pleasure by releasing DA in ludic situations and stimulating the body to seek a pleasant activity or occupation (Ayano, 2016).

#### 6.2.3 Mesocortical pathway

In the mesocortical pathway, dopaminergic fibers also arise from the VTA and project to the frontal cortex and septohippocampal regions. DA in this region regulates cognitive and emotional behavior. DA levels in the brain, especially in the prefrontal cortex, help improve working memory and attention (Björklund and Dunnett, 2007; Ayano, 2016).

#### 6.2.4 Tuberoinfundibular pathway

The tuberoinfundibular pathway originates in the arcuate nucleus of the hypothalamus (arcuate and paraventricular nuclei) and projects to the pituitary gland (the median eminence). In this pathway, DA inhibits prolactin release.

## 7 Evidence of alterations in the dopaminergic system associated with traumatic brain injury

### 7.1 Clinical studies in human subjects

Table 1 summarizes studies carried out in patients with TBI (mild, moderate, or severe) and alterations in the dopaminergic system at the central nervous system level. The approach taken in these includes (1) the dysfunction of the dopaminergic neuronal circuits and (2) modifications in the concentration of DA and its metabolites.

**Table 1:** Alterations of the dopaminergic system in patients with TBI.

Patients included in the study	Evaluation of TBI (GCS)	Evaluation and monitoring post-TBI	Dopaminergic system observations and clinical correlation	Additional information	Reference
30 subjects with TBI (19 men and 11 women) 21 healthy controls Age: 35.3 ± 10.8 years	20 mild 5 moderate 5 severe	MRI data were collected using a Siemens Prisma 3.0 Tesla MRI system. The volume of SN and the global functional connectivity of the SN and VTA was analyzed	Patients with TBI reported more anxiety and depressive symptoms. Smaller SN and reduced functional connectivity in the left SN, were seen in individuals with TBI.	Functional connectivity between left SN and left angular gyrus was positively associated with post-traumatic anxiety symptoms and negatively associated with depressive symptoms.	Gao L. et al. (2022)
36 patients with TBI (25 men and 11 women). Age: 41.0 ± 15.8 36 control subjects (25 men and 11 women) Age: 36.6 ± 9.3	GCS ≤ 12	Plasma adrenaline (A), noradrenaline (NA), and dopamine (DA) leves were determined	Patients with GCS 3 to 4 had markedly increase in baseline mean A, NA, and DA levels as compared with control, while patients with better GCS (11–12) had mildly elevated levels. GOS follow-up of 9.73 ± 2.26 months. Was negatively correlated with A and DA level at admission. No data are presented regarding the mood or anxiety in the patients.	Elevated A, NA and DA levels were an excellent marker that exhibit the extent of brain injury and predict the probability of recovery	Singh et al. (2022)
43 subjects with moderate to severe TBI Age: 39.7 ± 12.1	Mayo Ranking System	Single-Photon Emission ^123^Iioflupane DAT in caudate and putamen 5.94 years (0.5 to 30.5 years) post-TBI	All patients were in the chronic phase of TBI and presented cognitive difficulties. A decrease in DAT was observed mainly in the caudate nucleus, with involvement of the putamen, different from what was observed in patients in the early phase of Parkinson’s disease. No data are presented regarding the mood or anxiety in the patients.	TBI risk factor for development of Parkinson’s disease Identification of a hypodopaminergic state could allow clinical intervention. They consider that TBI, rather than giving a “static shock” to the dopaminergic system, produces an accelerated degeneration of the dopaminergic fibers.	Jenkins et al. (2020)
13 subjects with severe TBI Age: 18 to 50 years	GCS <9 Ten patients with severe TBI (77%) and 3 intubated or unknown severity (23%)	SPECT with ^123^Iioflupane at baseline and 2.5 h after a single dose of methylphenidate (enteral route) They measured the DAT in striatum and substantia nigra. 48 days post-TBI	Patients were evaluated at week 16 post TBI, with low cognitive functioning (Rancho Los Amigos ≤ IV) and moderately severe disability (Disability Rating Scale ≥7). Heterogeneous disruption of the nigrostriatal system was documented, and it was observed that the administration of methylphenidate is associated with a decrease in DAT density, which is important for a positive clinical response. No data are presented regarding the mood or anxiety in the patients.	^123^Iioflupane SPECT is a promising tool to determine the severity of the presynaptic DA terminal and to monitor the pharmacokinetics and pharmacodynamics of therapeutic interventions targeting the DA system.	Womack et al. (2020)
12 subjects with moderate to severe TBI: 6 with major depressive disorder (MDD) who had no history of depression before TBI. 6 without DDM 26 controls Age: 22 to 52 years	Mayo Classification System	D2/D3 in relation to major depression disorder and axonal pattern damage. PET (^11^C) PHNO, MRI, diffusion tensor imaging in caudate and tonsil 15 to 372 months post-TBI	Changes in D2/D3 receptors post-TBI, lower expression in the caudate (mainly in patients with MDD) and higher expression in the amygdala (patients without MDD). Abnormalities in white matter connections within the limbic system, with more extensive damage in patients with MDD	They suggest that compensatory changes in the dopaminergic system may be protective against the development of MDD after TBI	Jolly et al. (2019)
25 patients (14 M/11F) with persistent symptoms after a mild TBI; with one or more prior traumatic brain injuries and chronic symptoms Age: 51 ± 16 10 Control subjects (5 M/5F) Age: 57 ± 14	Patients had a history of at least one prior head injury over 6 months and continued symptoms.	SPECT with both ^99m^Tc Exametazime to measure cerebral blood flow (CBF) and ^123^Iioflupane to measure dopamine transporter (DAT) binding.	Higher striatum DAT binding was associated with more depressive symptoms	There was no clear association between CBF and DAT binding in these patients	Newberg et al. (2014)
12 subjects with TBI (adult men)	GCS: 7.3 ± 0.80 Moderate to Severe	PET Using [^11^C] βCFT for DAT and [^11^C] raclopride for D2R. The activity was analyzed in caudate, ventral striatum, putamen, and cerebellum. MRI 1-year post-TBI	Patients were included in the study 1 year after TBI, with executive function deficits as determined by neuropsychological testing. PET showed decreased binding to DAT in the caudate, putamen, and ventral striatum and increased D2R in the ventral striatum. Regarding the mood or anxiety in the patients, it is only mentioned that 4 of the subjects with TBI were taking antidepressants.	Results suggest that patients with TBI develop a hypodopaminergic state. Neuroimaging findings of TBI patients included diffuse axonal injury, hemorrhagic contusion, subdural hematoma, and subarachnoid hemorrhage.	Wagner et al. (2014)
63 patients with TBI (48 men and 15 women) Age: 31.49 ± 1.80	GCS < 8 severe TBI	HPLC was used to determine CSF levels of DA, DOPAC, and HVA during the first 5 days after injury. DAT patients’ genotypes were obtained using previously banked samples of CSF	DAT 10/10 genotype had higher CSF DA levels than patients with either the DAT 9/9 or DAT 9/10 genotypes. Females with the DAT 10/10 genotype had higher CSF DA levels than females with the DAT 9/9 or DAT 9/10 genotypes, and sex was associated with higher DOPAC levels. No data are presented regarding the mood or anxiety in the patients.	After severe TBI, patients’ CSF DA and HVA levels—but not DOPAC levels—were higher than reported control values	Wagner et al. (2007a)
10 subjects with TBI (8 men and 2 women) Age: 17 to 57 years (mean 33 ± 13)	5.8 ± 4.8 1 mild 1 moderate 8 severe	SPET (^123^I-β-CIT and ^123^I-IBZM). Semiquantitative activity of DAT and D2R by binding in the striatum. CCT MRI 141 ± 94.2 days post-TBI	The patients were in the chronic phase and presented motor alterations with akinetic-rigid characteristics. In the striatum, a 45% reduction in DAT binding and a 73% reduction in D2R were observed compared to control patients. No data are presented regarding the mood or anxiety in the patients.	CCT and MRI evaluations demonstrated cortical and subcortical lesions in all patients. Structural lesions were only found in the striatum of 1 patient. Alterations in the transporter and the receptor are independent of the severity of damage determined by GCS	Donnemiller et al. (2000)
48 patients with acute severe head injury Age 16 to 73 years, mean 40.15 ± 16.46 35 healthy volunteers of Ages 16 to 66 years, mean 35.74 ± 13.1	GCS ≤ 8	Serum A, NA and DA levels were measured at 3 and 7 days after injury. GCS was recorded on admission and GOS 1 month after injury.	DA levels were significantly higher in TBI compared to the control group. DA levels were not significantly related to outcome. No data are presented regarding the mood or anxiety in the patients.	Serum NA and A levels were higher in patients with lower GCS and in the group of patients who did not survive.	Yang et al. (1995)
15 patients with TBI (12 men and 3 women). Age: 33.5 ± 9.9	GCS of 8 or less	Daily measurements of urinary A, NA, metanephrine, normetanephrine, DA, and cortisol, and with plasma levels of insulin, glucagon, and C-reactive protein during 15 d.	Urinary A, NA, DA levels were significatively increased respect to controls. No data are presented regarding the mood or anxiety in the patients.	Urinary levels of norepinephrine, normetanephrine, and cortisol were higher in patients with lower GCS scores.	Feldman et al. (1993)
38 patients (34 men and 4 women). Age: 16 to 69	GCS scores during the first 48 h ranged from 3 to 13, with the majority of patients scoring below eight during this period.	Catecholamine Urinary levels of A, NA, DA were determinated.	Urinary DA levels remained within the normal range, and the increase noted in the first 7 days after injury continued, but was less marked for the survivors, and then declined again No data are presented regarding the mood or anxiety in the patients.	NA and DA levels increased with time since injury	Johnson et al. (1993)
24 patients in coma after head injury,	GCS 3–8 5.5 ± 1.7	Main metabolites of the neurotransmitters NA, DA, and serotonin, methoxyhydroxyphenylglycol (MHPG), homovanillic acid (HVA), and S-hydroxyindoleacetic acid (SHIAA) respectively, were estimated by HPLC with electrochemical detection in CSF samples	The group of patients had highly significant elevated concentrations of MHPG, SHIAA, and HVA compared with controls (*p* < 0.00 L), after removing the differences that can be accounted for age. No data are presented regarding the mood or anxiety in the patients.	The severity of the coma, as measured by the Glasgow Coma Scale, correlated only with the 5HIAA level	Markianos et al. (1992)
Patients: TBI (*n* = 24), vascular brain injury (*n* = 10), poly system trauma (*n* = 7) and medical/surgical patients in an intensive care unit (*n* = 29).		Blood samples were used to determine circulating free and total (free plus conjugated) NA, A, and DA	DA parameters remained normal. No data are presented regarding the mood or anxiety in the patients.	In the patients with TBI or vascular brain injury, significant inverse correlations were present between the degree of neurological dysfunction, as indicated by the GCS, and free and total NA, A, and DA levels	Woolf et al. (1988)
61 patients (50 male), aged 17 to 95 years (median 25 years), who had suffered traumatic brain injury within the preceding 48 h		Blood samples for NA, A, and DA measurement	Levels of NA, A, and DA correlated highly with the admission GCS score. No data are presented regarding the mood or anxiety in the patients.	In the 54 patients who survived beyond 1-week, significant correlations were present between the length of hospitalization and NA and A levels. Concentrations of NA and A were also correlated with the duration of ventilatory assistance.	Woolf et al. (1987)
33 patients with TBI (24 men and 9 women)	GCS: 3–11	Blood samples for determinations of NA; A and DA levels were obtained immediately upon arrival of the patient.	In patients with a GCS of 3 to 4, NA and A levels increased four-to fivefold and the DA level increased threefold above normal. No data are presented regarding the state of mood or anxiety in the patients	Circulating NA, A. and DA are excellent markers that reflect the extent of brain injury and that may predict the likelihood of recovery.	Hamill et al. (1987)
98 patients within 6 h after closed head injury.	72 patients were unconscious and 26 were conscious at the time of admission.	Concentrations of HVA and 5HIAA were measured in the lumbar cerebrospinal fluid.	The HVA levels decreased in patients, whether or not they were given probenecid, which inhibits the active transport of these acids from the brain. The decline of HVA was more notable in patients with the longest duration of unconsciousness. No data are presented regarding the state of mood or anxiety in the patients.	Results suggest a decreased cerebral DA and serotonin metabolism after head iniury.	Vecht et al. (1975)

GCS, Glasgow Coma Scale; A, adrenaline; NA, noradrenaline; DA, dopamine; MHPG, methoxyhydroxyphenylglycol; HVA, homovanillic acid; and 5-HIAA, 5-hydroxyindoleacetic acid.

#### 7.1.1 Dysfunction of dopaminergic neuronal circuits

The physiological changes in the dopaminergic system after TBI are closely linked to the direct mechanical damage of projection pathways. These pathways produce DA from brainstem nuclei, the retrorubral region (A8) of the VTA (A10), and the SNc (A9). These nuclei project towards the striatal, subcortical, and cortical regions via nigrostriatal, mesolimbic, and mesocortical pathways. Structural imaging suggests that TBI can disrupt these dopaminergic pathways, with magnetic resonance (MR) studies indicating that even 7 months after a moderate to severe TBI, patients show a diminished anisotropy index in most white matter tracts, axonal injury, and volume reduction of frontal lobes, striatum, and cerebellum, highlighting the long-term effects of TBI on the dopaminergic system.

Wagner et al. in 2014 reported decreased DAT and elevated expression of D2R in various brain regions, including the caudate, putamen, and ventral striatum, as determined using positron emission tomography (PET), in patients with moderate to severe TBI and executive function deficit, based on neuropsychological tests, suggesting the establishment of a hypodopaminergic state (Wagner et al., 2014). Jenkins et al. (2020) analyzed the alterations of the DAT, using single photon emission computed tomography (SPECT), in 43 patients with moderate to severe TBI in the chronic phase (more than 6 months from the lesion) and with cognitive difficulties. They found that patients with TBI present a decrease in DAT in the caudate nucleus, which has been related to hypodopaminergic states and motor alterations other than Parkinson’s disease in the early stages (Table 1).

On the other hand, an asymmetric depletion in DAT expression in the striatum 48 days after a moderate to severe traumatic brain injury has been reported and evaluated by SPECT, suggesting a heterogeneous disruption of the nigrostriatal pathway with low cognitive functioning and moderately severe disability (Womack et al., 2020; Table 1).

**Figure 2:**
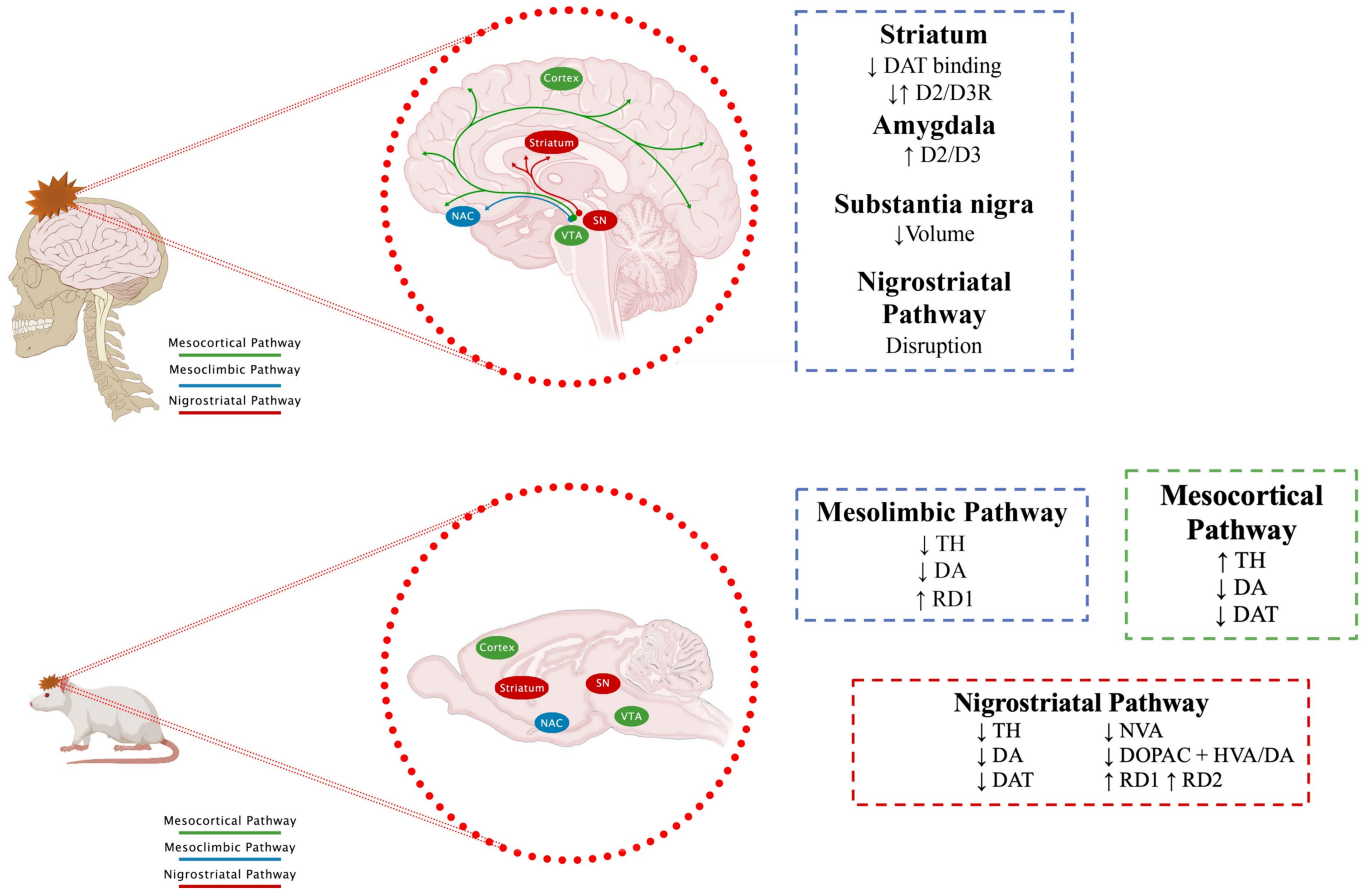
Effect of TBI on dopaminergic pathways. The main dopaminergic pathways and changes described after a TBI are shown. See text for further details. Section 7.1 for human data and section 7.2 for animal models.

In Donnemiller et al. (2000) (Table 1) determined the function of the DAT and the D2R in the striatum by SPECT and the presence of cortical and subcortical lesions by cranial computerized tomography (CCT) or MR in 10 patients with TBI (1 with Glasgow Coma Scale, GCS = 15, 1 with GCS = 12 and 8 with GCS less than or equal to 8), all in the chronic phase with an average time of 140.8 ± 94.2 days since the injury. On SPECT images, abnormal patterns of reduced tracer uptake were observed in the striatum in all patients, with an average 45% reduction in DAT binding and an average 73% reduction in D2R compared to controls. The degree of post-traumatic dysfunction of DAT and D2R in the striatum was unrelated to the severity of the injury initially assessed by GCS. Therefore, the authors concluded that the anatomical and functional alterations of the nigrostriatal or frontostriatal pathways could be more related to the clinical pictures presented by the patients, such as akinetic-rigid characteristics (Donnemiller et al., 2000); in this study, no alterations in behavior or mood disorders were reported; however, it was shown that, regardless of the GCS, there are alterations in the dopaminergic pathways in patients who have suffered a TBI but the depicted clinical pictures can also be the consequence of slow-onset and subtle events, such as receptor modifications, the interaction of DA with its receptors, changes in DAT, or the generation of sustained or alternating hypodopaminergic states (Figure 2).

#### 7.1.2 Changes in the concentration of DA and its metabolites

Furthermore, it is worth noting that several authors have reported alterations in the levels of DA and its metabolites in the biological fluids of patients post-TBI (Table 1). For example, Wagner et al. (2007a) studied 63 patients with severe TBI. They found elevated levels of DA and HVA in the patients in the 5 days following the TBI, which agrees with the study by Markianos et al. (1992), who had reported elevated levels of HVA in the cerebrospinal fluid (CSF) of 24 patients in coma after head injury, not like the report by Vecht et al. (1975), who found decreased HVA levels in patients sampled in the first hours post-trauma. Likewise, different reports in plasma from patients who have suffered a TBI indicate elevated levels of DA compared to controls (Hamill et al., 1987; Woolf et al., 1987, 1988; Yang et al., 1995; Singh et al., 2022). There are even some reports of increases in DA metabolites in the urine of patients who have suffered a TBI (Feldman et al., 1993; Johnson et al., 1993).

### 7.2 Animal model studies

Similarly, studies in animal models have reported that TBI induces an imbalance in dopaminergic transmission in brain regions that comprise dopaminergic pathways (Figure 2). Since, Edvinsson et al. (1971) reported a significant decrease in DA levels in the brains of rabbits after 48 h of inducing a TBI by inserting a small pressure cannula into the brain (Edvinsson et al., 1971). Subsequent studies have reported changes in specific regions. For example, in SN (Table 2), the predominant findings show reductions in TH levels, regardless of whether the model used was a rat (Hutson et al., 2011; Sauerbeck et al., 2012; Van Bregt et al., 2012; Acosta et al., 2015; Tan et al., 2015; Liu et al., 2017) or mouse (Selvakumar et al., 2020; D'Amico et al., 2021), this decrease in TH is observed from one to 78 days after TBI. Only Schmidt and Grady (1995), who did not do a quantitative analysis, did not find changes in TH. Yan, who used a severe TBI, reported a delayed increase in TH 28 days after TBI, interpreting it as a possible compensatory response (Yan et al., 2007). Despite the changes described in TH, no significant changes were reported in DA. Regarding some DA metabolites such as HVA, Van Bregt et al. (2012) reports a decrease, while Liu et al. (2017) find an increase. In all cases, immunohistochemistry was used to analyze TH; some studies even use stereology and/or WB for better quantification. The sample sizes (3 to 10) are those commonly reported in these studies.

**Table 2:** Alterations of the dopaminergic system in substantia nigra secondary to TBI in rodent models.

Animal species Bodyweight, Age,	Experimental model	TBI severity	Time post TBI	Dopaminergic system changes	Evaluation of depressive behavior or anxiety	Reference
Adult male Sprague–Dawley rats, 275–325 g (*n* = 4–5)	FP	Mild to moderate	11–15 d	=TH (Immunohistochemistry) (It has not been quantitatively analyzed)	Not reported	Schmidt and Grady (1995)
72 male Sprague–Dawley rats 250–275 g	CCI	Severe	1, 7 d 28 d	=TH ↑TH (Immunohistochemistry, WB) (*n =* 6)	Not reported	Yan et al. (2007)
63 adult Male Sprague-Dawley rats 2–3 months	FP	Moderate	11, 182 d	↓TH (Immunohistochemistry) (*n =* 6–10)	Not reported	Hutson et al. (2011)
Adult Male Sprague-Dawley rats 350–375 g	FP	Moderate	28 d	= DA, ↓HVA, ↓ (DOPAC + HVA/DA) (HPLC-ECD) (*n =* 5) ↓TH (Immunohistochemistry-stereology) (*n =* 3)	Not reported	Van Bregt et al., 2012
Male Fischer 344 rats 16 weeks old	CCI	Mild and moderate	30 d	↓TH (Immunohistochemistry) (*n =* 6)	Not reported	Sauerbeck et al. (2012)
Male Sprague-Dawley rats Ten-week old (*n =* 12; sham or TBI).	CCI	Not reported	60 d	↓TH (Immunohistochemistry-stereology)	Not reported	Acosta et al. (2015)
Adult Male Sprague-Dawley rats 250–280 g	CCI	Not reported	7 d	↓TH (Immunofluorescence) (*n =* 8)	↓ Sucrose preference (*n =* 8) ↑Immobility time in FST (*n =* 8)	Tan et al. (2015)
Male CD1 mice (25 to 30 g) 10–12 weeks (*n =* 10)	CCI	Not reported	30 d	↓TH, ↓DAT (Immunohistochemistry, WB) (*n =* 5)	↓Time in the open arm of EPM (*n =* 10)	Impellizzeri et al. (2016)
Adult male Sprague–Dawley rats	FP	Moderate	28 d	↓TH =DA, =DOPAC ↑HVA (Immunohistochemistry-stereology; HPLC-ECD) (*n =* 6)	Not reported	Liu et al. (2017)
Male Sprague–Dawley rats, weighing 90 ± 5 g (4 weeks old) (*n =* 10 in each group)	CCI	Not reported	48 d	↓TH y ↑R2D (Immunohistochemistry, WB)	Not reported	Ko et al. (2019)
C57BL/6 mice (25 ~ 30 gm), 10 ~ 12 weeks	CHI	Mild	24, 72 h	↓TH (Immunofluorescence, WB) (*n =* 6)	↑Immobility time in TST (*n =* 6)	Selvakumar et al. (2020)
Male CD1 mice (25 to 30 g) (10 mice for each group)	CCI	Not reported	30 d	↓TH ↓DAT (Immunohistochemistry) (*n =* 5)	↓Time in open arm EPM (*n =* 10)	D'Amico et al. (2021)

FP, fluid percussion; CCI, controlled cortical impact; CHI, closed head injury; TH, tyrosine hydroxylase. DA, dopamine; HVA, homovanillic acid; DOPAC, dihydroxyphenylacetic acid; DAT, DA transporter; RD2, DA receptor type 2; HPLC-ECD, high performance liquid chromatography-electrochemical detection; WB, western blot; FST, forced swimming test; EPM, elevated plus maze; ↑, increases. ↓, reduces, =, not changed.

Regarding the striatum, it is worth highlighting that there are many more reports (Table 3). Some authors report an increase in DA in the first hours after injury (McIntosh et al., 1994; Massucci et al., 2004; Muthuraju et al., 2014), while in chronic stages, the release and levels of DA decrease (Shin et al., 2011; Huang et al., 2014), as well, TH diminish its expression as the injury time elapses (McIntosh et al., 1994; Massucci et al., 2004; Wagner et al., 2005a; Shin et al., 2011; Huang et al., 2014; Chen et al., 2015; Tan et al., 2015). Also, it has been reported that after a TBI, there is a significant decline of DAT in the striatum (Wagner et al., 2005a,b; Karelina et al., 2017), a decrease or no change of HVA and DOPAC (Shin et al., 2011; Chen et al., 2015). It is also worth noting that the changes observed depend on the magnitude of the damage. In this sense, Chen found a significant decrease in DA release in mild and severe injuries; However, this decrease is temporary in mild injuries (Chen et al., 2015).

**Table 3:** Alterations of the dopaminergic system in the striatum secondary to TBI in rodent models.

Animal species Bodyweight, Age,	Experimental model	TBI severity	Time post TBI	Dopaminergic system changes	Evaluation of depressive behavior or anxiety	Reference
60 male Sprague–Dawley rats (weighing 300–350 g	FP	Moderate	6 h, 1, 24 h, 7, 14 d	↑ DA = DA (HPLC) (*n =* 6)	Not reported	McIntosh et al. (1994)
Adult male Sprague–Dawley rats, 275 to 325 g (*n =* 4–5)	FP	Mild to moderate	11–15 d	=TH (Immunohystochemistry)	Not reported	Schmidt and Grady (1995)
100 male Sprague–Dawley rats 300–325 g	CCI	Not reported	1-h 1, 7, 14, 28 d	↑DA, ↑DOPAC = DA, = DOPAC (HPLC-ECD) (*n =* 10)	Not reported	Massucci et al. (2004)
Adult male Sprague–Dawley rats 300–330 g	CCI	Severe	14, 28 d	↓DAT (WB) (*n =* 6)	Not reported	Wilson et al. (2005)
Adult Male Sprague- Dawley rats (19 CCI and 16 ctrl)	CCI	No incidents of mortality following	14 d	↓ Evoke DA overflow ↓ DA clearence (FSCV) (*n =* 7–10) ↓DAT, = VMAT, = DR2 (WB) (*n =* 3)	Not reported	Wagner et al. (2005a)
Male and female Sprague–Dawley rats Male (*n =* 24) and cycling female (*n =* 24)	CCI	Not reported	28 d	=DAT (females) ↓DAT (males) (WB) (*n =* 6)	Not reported	Wagner et al. (2005b)
72 male Sprague–Dawley rats 250–275 g	CCI	Severe	1, 7 d 28 d	=TH ↑TH (Immunohistochemistry, WB) (*n =* 6)	Not reported	Yan et al. (2007)
Young adult male, Sprague–Dawley rats CCI injury (*n =* 26) or were naïve controls (*n =* 26).	CCI	Not reported	15 d	↓DA release and clearance ↓DAT =TH, =DR1, =DR2 = VMAT (FSCV (*n =* 7–8), WB (*n =* 6))	Not reported	Wagner et al. (2009)
Adult male CD1 mice 1 (*n =* 28) and 36 h (*n =* 28)	CHI	Mild	1 h, 36 h	=DA, ↓DOPAC/DA ↑DA, ↓DOPAC/DA (HPLC-ECD) (*n =* 7)	Not reported	Shen et al. (2011)
63 adult Male Sprague-Dawley rats 2-3 months	LFP	Moderate	11, 182 d	↓TH (Immunohistochemistry) (*n =* 4–7)	Not reported	Hutson et al. (2011)
Male Sprague- Dawley rats 280–350 g	CCI	Severe	1 d 7 d 28 d	= DA-release K+ stimulated = DOPAC, HVA = TH activity, pser40TH ↓DA release K+ stimulated = DOPAC, HVA ↓ TH activity, pser40TH = DA-release K+ stimulated = DOPAC, HVA ↓ TH activity, = pser40TH (Microdialysis-HPLC-ECD (*n =* 7–10); enzimatic activity (*n =* 6); WB (*n =* 6))	Not reported	Shin et al. (2011)
Male Fischer 344 rats 16 weeks old	CCI	Mild and moderate	30 d	=D2R, =DAT (WB) (*n =* 6)	Not reported	Sauerbeck et al. (2012)
96 C57BL/J6 male mice 25 and 30 g	FP	Severe	1, 7, 14, 21, 28 d	↓DR1, ↓DR2, ↓DAT and ↓VMAT (Immunohistochemistry) (n, not reported)	Not reported	Abdullah et al. (2013)
130 young adult male Sprague–Dawley (6-week old) 200–250 Sham, *n =* 5, mild, *n =* 15, high, *n =* 1,	FP	Mild and severe	7 14, 28, 42, 56 d 2, 24 h; 7, 14, 56 d	↓ DA release (severe TBI) =DA, =HVA + DOPAC/DA ↑HVA + DOPAC/DA (FSCV) (*n =* 3–4); (HPLC-ECD) (*n =* 5–13)	Not reported	Huang et al. (2014)
C57BL/6 J mice	CHI	Moderate	14 d	↑pDARPP-32 (WB) (*n =* 3–4)	Not reported	Lowing et al. (2014)
Male imprinting control region (ICR) mice (25–30 g)	CHI	Mild	1, 7, 30 d 24 h	↑TH ↑D2R binding WB (*n =* 6–8), radiolabeling	Not reported	Edut et al. (2014)
60 adult male C57BL/J6 mice (25–30 g)	FP	Severe	3 h 6, 12 h 24 h	↓DA ↑DA =DA (ELISA)	Not reported	Muthuraju et al. (2014)
Male Sprague–Dawley rats 250–280 g	CCI	Not reported	3 h 6, 12, 24 h; 3,7 d	↑DA, DOPAC, HVA ↓DA, DOPAC, HVA (HPLC-ECD) (*n =* 8)	↓ Sucrose preference (*n =* 8) ↑Immobility time in FST (*n =* 8)	Tan et al. (2015)
Young adult male Sprague–Dawley rats (mild TBI, *n =* 50; severe TBI, *n =* 51; sham, *n =* 21).	FP	Mild and severe	2, 24 h 7, 14, 28, 42, 56 d	↓ DA release (severe TBI); = DA, = DOPAC + HVA/DA =TH FSCV (*n =* 3); HPLC-ECD (*n =* 3); WB	Not reported	Chen et al. (2015)
Young adult (2–3 months) CD-1 male and female mice	CCI	Not reported	1 d	↓DA (males and females) ↓ K+ stimulated DA release (females) ↓DAT mRNA, (females) ↓VMAT-2 mRNA (females) =DAT (males); ↓DAT (females) HPLC (*n =* 6–12), *In vitro* superfusion (*n =* 12); RT-PCR (*n =* 4); WB (*n =* 4)	Not reported	Xu et al. (2016)
Female Swiss Webster mice 21 d	CHI	Mild	49 d	=TH, ↓DAT, ↓D2R (Immunohistochemistry) (*n =* 7–9)	Not reported	Karelina et al. (2017)
Adult male Sprague–Dawley rats weighing 325–425 g	CCI	Not reported	7 d	↓DA release =pSer40-TH (synaptosomes), =D2R (synaptosomes), =DAT (synaptosomes) (Microdialysis-HPLC-ECD (*n =* 6);WB (*n =* 6))	Not reported	Carlson and Dixon (2018)
Male Sprague–Dawley rats, weighing 90 ± 5 g (4 weeks) (*n =* 10 in each group)	CCI	Not reported	48 d	↓TH y ↑R2D (Immunohistochemistry, WB)	Not reported	Ko et al. (2019)
68 male Long-Evans rats 3 months Severe TBI (*n =* 28), Mild TBI (*n =* 23), or Sham (*n =* 17)	CCI	Mild and severe	25 d	↓D1R, = D2R, = DAT, (WB)	Not reported	Vonder Haar et al. (2019a)
Male Wistar rats 4–5 months (7 animals/group except control) (*n =* 5).	CHI	Not reported	29 d	↓DA (HPLC-ECD)	Not reported	Rana et al. (2020)

FP, fluid percussion; CCI, controlled cortical impact; CHI, closed head injury; TH, tyrosine hydroxylase. DA, dopamine; HVA, homovanillic acid; DOPAC, dihydroxyphenylacetic acid; DAT, DA transporter; RD2, DA receptor type 2; pser40TH, ser40-phosphorylated tyrosine hydroxylase; VMAT, vesicular monamine transporter; FSCV, fast scan cyclic voltammetry; ELISA, enzyme-linked immuno sorbent assay; EIA, enzyme immunoassay; HPLC HPLC-ECD, high performance liquid chromatography-electrochemical detection; WB, RT-PCR, reverse transcription & polymerase chain reaction. Western blot; FST, forced swimming test; EPM, elevated plus maze; ↑, increases. ↓, reduces, =, not changed.

It is worth noting that, in general, HPLC-ECD was used to report DA levels, and the sample sizes referred to are those generally reported in these studies. However, some authors find increases in DA in the first hours post-TBI, and others find decreases.

In the cerebral cortex (Table 4), there is greater diversity in the reports. Some authors find an increase in DA levels, not only in the acute phase (Massucci et al., 2004) but also in the chronic phase (Kobori et al., 2006; Tanaka et al., 2019); likewise, some authors report that there are no changes in DA levels (Tsuda et al., 2020; Hu et al., 2023) or even find decreases (McIntosh et al., 1994; Rana et al., 2020). Similarly, there are reports of an increase in TH (Kobori et al., 2006), a decrease (Edut et al., 2014), or no changes (Kaukas et al., 2021). Yan et al. (2001) using a model of controlled cortical impact (CCI) in Male Sprague–Dawley rats, did not find changes in TH expression in the first 14 days but observed an increase at 28 days (Yan et al., 2001). Once again, it is worth noting that the results reported are not homogeneous despite using the same analysis techniques. In particular, in this table, the observations also depend on the subregion of the cerebral cortex analyzed.

**Table 4:** Alterations of the dopaminergic system in cerebral cortex secondary to TBI in rodent models.

Animal species Bodyweight, Age,	Experimental model	TBI severity	Time post TBI	Dopaminergic system changes	Analyzed region	Evaluation of depressive behavior or anxiety	Reference
60 male Sprague–Dawley rats (300–350 g)	FP	Moderate	1, 6, 24 h, 7, 14 ds	↓DA (HPLC) (*n =* 6)	Parietal cortex	Not reported	McIntosh et al. (1994)
70 male ICR mice 28–30 g	CHI	Not reported	25 s, 3.5 m	= DA, =DOPAC+HVA/DA (HPLC-ECD) (*n =* 3–6)	Telencephalon without the ‘striatum’	Not reported	Tanaka et al. (1997)
96 male Sprague-Dawley rats 250–275 g	CCI	Not reported	1, 7, 14 d 28 d	= TH, =DBH, =DAT ↑TH ↓DAT, =DBH (Immunohistochemistry, WB) (*n =* 6)	Frontal cortex	Not reported	Yan et al. (2001, 2002)
100 male Harlan Sprague–Dawley rats 300–325	CCI	Not reported	1-d 7, 14, 28 d	↑DA, ↑DOPAC = DA, = DOPAC (HPLC-ECD) (*n =* 10)	Frontal cortex	Not reported	Massucci et al. (2004)
Male and female Sprague-Dawley rats Male (*n =* 24) and Female (*n =* 24)	CCI	Not reported	28 d	=DAT (females) ↓DAT (males) (WB) (*n =* 6)	Frontal cortex	Not reported	Wagner et al. (2005b)
Male Sprague-Dawley rats	CCI	Moderate	14 d 28 d	↑TH = DA =TH ↑DA (WB, EIA) (*n =* 5)	Prefrontal cortex	Not reported	Kobori et al. (2006)
Male imprinting control region (ICR) mice 25–30 g,	CHI	Mild	24 h, 1 h, 3, 7, 30 d	↓TH =TH (WB) (*n =* 6–8)	Whole cerebral cortex	Not reported	Edut et al. (2014)
Neonatal Sprague–Dawley rat	repeated shaking brain injury	Mild	56–70 d	↑DA; =DOPAC (HPLC) (*n =* 7–10)	Dorsal part of the medial prefrontal cortex	↓ time in open arm (EPM) (*n =* 35)	Tanaka et al. (2019)
Male Sprague–Dawley rats, weighing 90 ± 5 g (4 weeks) (*n =* 10 in each group)	CCI	Not reported	48 d	↓TH y ↑R2D (Immunohistochemistry, WB)	Prefrontal cortex	Not reported	Ko et al. (2019)
Male Wistar rats 4–5 months 7 animals/group except control, *n =* 5).	CHI	Not reported	29 d	↓DA (HPLC-ECD)	Whole cerebral cortex	Not reported	Rana et al. (2020)
Adult male Sprague–Dawley rats (12–14 weeks old, 250–280 g) (*n =* 7 per group)	Repetitive blast-induced traumatic brain injury (bTBI)	Mild	2 d after the last bTBI	=DA (HPLC-ECD)	Motor cortex	Not reported	Tsuda et al. (2020)
Male and female Sprague Dawley rats. Animals at p35	CHI	Injury confirmed by a significant increase in righting reflex	49 d	=TH, =DAT (WB) (*n =* 5–7)	Prefrontal cortex	Not reported	Kaukas et al. (2021)
Sprague Dawley rats (220–240 g, 8–9 weeks)	CCI	Severe	14 d	=DA (ELISA) (*n =* 3)	Prefrontal cortex	Not reported	Hu et al. (2023)

FP, fluid percussion; CCI, controlled cortical impact; CHI, closed head injury; TH, tyrosine hydroxylase. DA, dopamine; HVA, homovanillic acid; DOPAC, dihydroxyphenylacetic acid; DAT, DA transporter; RD2, DA receptor type 2; pser40TH, ser40-phosphorylated tyrosine hydroxylase; VMAT, vesicular monamine transporter; FSCV, fast scan cyclic voltammetry; ELISA, enzyme-linked immuno sorbent assay; EIA, enzyme immunoassay; HPLC HPLC-ECD, high performance liquid chromatography-electrochemical detection; WB, RT-PCR, reverse transcription & polymerase chain reaction. Western blot; FST, forced swimming test; EPM, elevated plus maze; ↑, increases. ↓, reduces, =, not changed.

Regarding NAc (Table 5), Chen et al. (2017a, 2018) find a decrease in DA release and reuptake in the core and shell and more significant changes in the core, while Huang et al., 2014 do not find differences in total DA. Regarding TH, there is a report of an increase in its expression at 49 days post-TBI (Kaukas et al., 2021), and concerning the expression of D1R, Bhowmick et al., 2021 describe a decrease at 2 days post-TBI, while Vonder Haar et al., 2019a finds an increase after 25 days.

**Table 5:** Alterations of the dopaminergic system in the nucleus accumbens secondary (NAc) to TBI in rodent models.

Animal species Bodyweight, Age,	Experimental model	TBI severity	Time post TBI	Dopaminergic system changes	Evaluation of depressive behavior or anxiety	Reference
Male Sprague–Dawley rats (6-week old) 200–250 g Sham, *n =* 5; mild, *n =* 15; severe *n =* 15.	FP	Mild and severe	2, 24 h; 7, 14, 28 d	=DA, ↑HVA + DOPAC/DA (HPLC-ECD) (*n =* 5–13)	Not reported	Huang et al. (2014)
Young adult male, Sprague-Dawley rats (6 weeks old) 200–250 g Sham, *n =* 28, mild, *n =* 42; severe, *n =* 42.	FP	Mild and severe	2, 24 h 7, 14, 28, 42, 56 d	↓DA release and reuptake core and shell; greater changes in the core. More evident 1–2 weeks in post TBI severe (FSCV) (*n =* 14)	Not reported	Chen et al. (2017a)
Male Sprague-Dawley rats	LFP	Moderate	11 d	↓DA release (FSCV) (*n =* 12)	Not reported	Chen et al. (2018)
68 male Long-Evans rats Sham, *n =* 17; mild, *n =* 23; severe, *n =* 28.	CCI	Mild and severe	25 d	↑D1R (mild TBI), =D2R, =DAT (WB)	Not reported	Vonder Haar et al. (2019a)
Male and female Sprague Dawley rats. Animals at p35	CHI	Injury confirmed by a significant increase in righting reflex	49 d	↑TH, ↑DAT (males) (WB) (*n =* 5–7)	Not reported	Kaukas et al. (2021)
Male C57/BL6 9 weeks old, 20–25 g;	FP	Mild and moderate	2 d	↓ D1R (WB) (*n =* 6)	↓ sucrose preference (*n =* 6)	Bhowmick et al. (2021)

FP, fluid percussion; CCI, controlled cortical impact; CHI, closed head injury; TH, tyrosine hydroxylase. DA, dopamine; HVA, homovanillic acid; DOPAC, dihydroxyphenylacetic acid; DAT, DA transporter; RD2, DA receptor type 2; pser40TH, ser40-phosphorylated tyrosine hydroxylase; VMAT, vesicular monamine transporter; FSCV, fast scan cyclic voltammetry; ELISA, enzyme-linked immuno sorbent assay; EIA, enzyme immunoassay; HPLC HPLC-ECD, high performance liquid chromatography-electrochemical detection; WB, RT-PCR, reverse transcription & polymerase chain reaction. Western blot; FST, forced swimming test; EPM, elevated plus maze; ↑, increases. ↓, reduces, =, not changed.

Concerning other regions analyzed (Table 6), there are only a few reports, for example, in the brainstem or specifically the locus coeruleus (LC), where no changes in DA are reported (McIntosh et al., 1994; Tsuda et al., 2020), or in VTA, where there is only one report in which a decrease in TH is referred, 49 days after the TBI (Karelina et al., 2017). Another less studied region is the hippocampus, where some authors report that there are no changes in DA levels (Tsuda et al., 2020) or it decreases (Hu et al., 2023), while in the hypothalamus, there are reports of increased DA (McIntosh et al., 1994).

**Table 6:** Alterations of the dopaminergic system in some cerebral areas secondary to TBI in rodent models.

Brain region	Animal species Bodyweight, Age,	Experimental model	TBI severity	Time post TBI	Dopaminergic system changes	Evaluation of depressive behavior or anxiety	Reference
Brainstem	60 male Sprague–Dawley rats (weighing 300–350 g)	FP	Moderate	1, 6, 24 h, 7, 14 d	=DA (HPLC) (*n =* 6)	Not reported	McIntosh et al. (1994)
Locus coeruleus	Adult male Sprague–Dawley rats (12–14 weeks old, 250–280 g) (*n =* 7 per group)	Repetitive blast-induced TBI	Mild	2 d after the last bTBI	=DA (HPLC-ECD)	Not reported	Tsuda et al. (2020)
Retrotuberal field	40 male C57BL/6 J mice 21–29 g	CCI	Not reported	1, 7, 14 d 4 d	↓DAT mRNA ↑DAT mRNA (*In situ* hybridization) (*n =* 5)	Not reported	Shimada et al. (2014)
VTA	Female Swiss Webster mice 21 d	CHI	Mild	49 d	↓TH, = DAT (Immunohistochemistry) (*n =* 7–9)	Not reported	Karelina et al. (2017)
Hypothalamus	60 male Sprague–Dawley rats (300–350 g)	FP	Moderate	1, 6 h 24 h, 7, 14 d	↑ DA = DA (HPLC) (*n =* 6)	Not reported	McIntosh et al. (1994)
Hippocampus	Adult male Sprague–Dawley rats (12–14 weeks old, 250–280 g) (*n =* 7 per group)	Repetitive blast-induced TBI	Mild	2 d after the last bTBI	= DA (HPLC-ECD)	Not reported	Tsuda et al. (2020)
	Sprague Dawley rats (220–240 g, 8–9 weeks)	CCI	severe	14 d	↓DA (ELISA) (*n =* 3)	Not reported	Hu et al. (2023)

FP, fluid percussion; CCI, controlled cortical impact; CHI, closed head injury; TH, tyrosine hydroxylase. DA, dopamine; HVA, homovanillic acid; DOPAC, dihydroxyphenylacetic acid; DAT, DA transporter; RD2, DA receptor type 2; pser40TH, ser40-phosphorylated tyrosine hydroxylase; VMAT, vesicular monamine transporter; FSCV, fast scan cyclic voltammetry; ELISA, enzyme-linked immuno sorbent assay; EIA, enzyme immunoassay; HPLC HPLC-ECD, high performance liquid chromatography-electrochemical detection; WB, RT-PCR, reverse transcription & polymerase chain reaction. Western blot; FST, forced swimming test; EPM, elevated plus maze; ↑, increases. ↓, reduces, =, not changed.

Although the animal models described in Tables 2–6 are rats and mice, it is worth noting that Kumar and Singh, using a TBI model in *Drosophila melanogaster* (fruit flies), found a decrease in DA levels 24 h post-TBI (Kumar and Singh, 2023).

## 8 Potential mechanisms linking TBI mood and anxiety disorders to alterations in the dopaminergic system

As shown in the previous sections, extensive literature validates the alterations of the dopaminergic system after TBI (Tables 1–6) as well as the association of TBI with mood disorders and anxiety (Whelan-Goodinson et al., 2009; Bryant et al., 2010; Guillamondegui et al., 2011; Osborn et al., 2014; Jorge, 2015; Perry et al., 2016; Juengst et al., 2017; Abdullah et al., 2018; Laliberté Durish et al., 2018; Robert, 2020; Ryttersgaard et al., 2020; Eliasen et al., 2021; Sameh et al., 2021). Likewise, several reports indicate that deregulation of the dopaminergic system may be one of the mechanisms for the development of mood and anxiety disorders (Nestler and Carlezon, 2006; Dunlop and Nemeroff, 2007.

In this section, we outline and underscore the potential biological pathways, neuronal circuits, and molecular mechanisms that could be involved in mood and anxiety disorders following TBI and their association with dopaminergic system alterations.

### 8.1 Impact of TBI on dopaminergic pathways

#### 8.1.1 Nigrostriatal pathway

TBI often damages this pathway, leading to motor deficits and cognitive dysfunction. Inflammation and oxidative stress can reduce DA levels and alter receptor density in the striatum, contributing to Parkinsonism-like symptoms (Dunlop and Nemeroff, 2007; Gao L. et al., 2022). In this sense, it is worth highlighting the recent studies by Gao L. et al. (2022) (Table 1), who reported a decrease in the SN volume of patients who had suffered TBI. They used MR to analyze patients with mild, moderate, and severe TBI who reported anxiety and depressive symptoms. They found a decrease in the size of the SN, particularly the left SN, and reduced functional connectivity in the left SN. The functional connectivity between the left SN and left angular gyrus was positively associated with anxiety symptoms and negatively associated with depressive symptoms (Gao L. et al., 2022). Also, there is evidence in rodent models suggesting that the decreased DA levels after TBI in different brain regions are closely related to the appearance of depression-like behavior; there are reports describing that after a controlled cortical impact or fluid percussion, the preference for sucrose consumption of animals is diminished, and the time of immobility recorded in the forced swim test increases; these behavioral data correlate with altered DA levels in the rat striatum (Nestler and Carlezon, 2006; Tan et al., 2015). Alternatively, the studies by Impellizzeri et al. (2016) and D'Amico et al. (2021) described a decrease in TH and DA in the SN and midbrain associated with a decrease in the time in the open arm of the Elevated plus maze.

#### 8.1.2 Mesolimbic and mesocortical pathways

The brain nuclei composing the mesocortical and the mesolimbic pathways play a predominant role in reward systems (Nestler and Carlezon, 2006; Turner et al., 2007; Heshmati and Russo, 2015), major depression (Heshmati and Russo, 2015), Parkinson’s disease (Wen et al., 2013) and chronic stress (Tye et al., 2013). These nuclei are highly susceptible to the effects of TBI, and their dysregulation can lead to anhedonia, decreased motivation, and executive dysfunction, which are common in post-TBI depression and anxiety (Dunlop and Nemeroff, 2007; Gao L. et al., 2022).

The mesolimbic pathway’s involvement in reward and motivation is crucial for understanding TBI-induced depression. Reduced DA signaling in the NAc can lead to anhedonia, a core symptom of depression. TBI patients may exhibit decreased responsiveness to rewarding stimuli and reduced motivation to engage in previously pleasurable activities (Dunlop and Nemeroff, 2007; Gao L. et al., 2022).

It has been suggested that the mesolimbic dopaminergic system, especially VTA, which projects and releases DA to NAc, plays a fundamental role in the development of anxiety disorder (Small et al., 2016). In support of the above, one study reported that after TBI induced by repeated bursts in mice, DA release increased in the NAc for 30 days after injury (Schindler et al., 2017); in addition, another study reported that a mild TBI generated by repeated impacts using rotational acceleration induced an alteration in the mesolimbic reward system by decreasing TH immunoreactivity in the olfactory tubercle (Vonder Haar et al., 2019b). Meanwhile, Tanaka et al., 2019, reported an increase in DA in the prefrontal cortex associated with a decrease in the time in the open arm of the Elevated plus maze.

Furthermore, it is worth emphasizing that the executive dysfunction often observed in patients with TBI highlights the role of the mesocortical pathway in cognitive functions. Disrupted dopamine signaling in the prefrontal cortex can lead to deficits in attention, working memory, and cognitive flexibility, contributing to the overall burden of psychiatric symptoms following traumatic brain injury.

#### 8.1.3 Hypothalamic-pituitary-adrenal axis

The interaction between dopaminergic and stress-responsive systems, such as the HPA, is significant in TBI. Dysregulated DA signaling can alter the stress response, leading to heightened anxiety and stress sensitivity. Elevated cortisol levels typical in TBI patients can further impair dopaminergic function, exacerbating anxiety symptoms (Dunlop and Nemeroff, 2007; Gao L. et al., 2022).

Murine models have shown that anxiety levels increase after a traumatic brain event, as the exploration time in the open field test and the permanence in the open arms of the elevated cross maze test decreases, as compared to control groups; together, with a considerable increase in serum corticosterone levels that are indicative of increased anxiety as a result of TBI (Fromm et al., 2004; Sönmez et al., 2007; Wagner et al., 2007b; Jones et al., 2008; Baykara et al., 2012, 2013; Almeida-Suhett et al., 2014; Broussard et al., 2018; Beitchman et al., 2020).

### 8.2 Molecular mechanisms

#### 8.2.1 DA synthesis and release

TBI can impair DA synthesis and release. Oxidative stress and inflammation may reduce TH, the rate-limiting enzyme in DA synthesis. Mitochondrial dysfunction post-TBI decreases ATP production, further impairing DA synthesis and release (Dunlop and Nemeroff, 2007; Gao L. et al., 2022). However, direct measurements of DA in biological fluids of patients post-TBI describe an increase in DA in the acute phase. For example, Wagner et al. (2007a) found increased levels of DA in CSF during the first 5 days after severe injury. However, already in 2014, using PET studies, the same author suggested that patients develop a hypodopaminergic state 1 year after trauma (Wagner et al., 2014). Regarding studies in experimental models of TBI, very few describe alterations in the dopaminergic system and include evaluations of depressive behavior or anxiety. These describe decreases in TH in SN associated with depression-like behavior (Tan et al., 2015; Selvakumar et al., 2020) or anxiety (Impellizzeri et al., 2016; D'Amico et al., 2021) as well as a decrease in DA levels in the striatum (Tan et al., 2015), or an increase in DA in the prefrontal cortex associated with “anxiety” type behaviors (Tanaka et al., 2019).

#### 8.2.2 Receptor dysregulation

Post-TBI, changes in DA receptor density and sensitivity are common. Upregulation of D2 receptors in response to decreased DA availability can lead to increased sensitivity to dopaminergic drugs and altered signaling, contributing to psychiatric symptoms (Dunlop and Nemeroff, 2007; Jolly et al., 2019). However, this appears to be region-specific. In Jolly et al. (2019) (Table 1) studied in moderate to severe TBI patients the relationship between post-traumatic depression, manifested as major depression disorder (MDD) and evaluated the availability of D2R/D3R and the microstructure of white matter. They observed that all patients with TBI had a decreased availability of receptors in the caudate and thalamus compared with the control groups, and this decrease was more significant in the TBI group with MDD.

In contrast, the TBI group without MDD presented a greater availability of the amygdala receptors than the control group. Another result was that the group with TBI and MDD presented more significant evidence of axonal damage compared to the TBI group without MDD in tracts that connect the limbic system. Changes in D2R and D3R receptors have also been reported, with differential expression in the caudate and amygdala and abnormalities in the limbic system, suggesting that compensatory changes in the dopaminergic system may be protective against the development of post-TBI major depression (Jolly et al., 2019). Regarding studies in experimental models of TBI, only one article describes a decrease in D1R in the nucleus accumbens associated with depression-like behavior (Bhowmick et al., 2021). Some authors report D2R changes in various brain regions but do not carry out tests for depression or anxiety-type behaviors.

#### 8.2.3 Transporter and metabolism alterations

DAT function may be compromised post-TBI, impairing DA reuptake, and altered synaptic DA levels. Changes in MAO and COMT activites can further dysregulate DA signaling (Dunlop and Nemeroff, 2007; Gao L. et al., 2022).

Newberg et al. (2014) (Table 1), reported that higher striatum DAT binding, evaluated by SPECT, was associated with more depressive symptoms, analyzing a sample of patients who had suffered a mild TBI over 6 months and continued symptoms (Newberg et al., 2014).

In experimental TBI models, DAT expression decreases have been reported in SN (Impellizzeri et al., 2016; D'Amico et al., 2021) and striatum (Wilson et al., 2005) associated with anxiety-type behaviors. Additionally, various authors have reported decreased DAT expression in the striatum from 1 day to 28 days post-TBI.

### 8.3 Insights from pharmacological treatments

Regarding pharmacological treatments for depression after traumatic brain injury, recent reviews agree that there is insufficient evidence to recommend prescribing any specific drug or drug (Plantier et al., 2016; Hicks et al., 2023). However, they also suggest pharmacological treatment may be effective in reducing depressive symptoms in those with depression following TBI (Slowinski et al., 2019), considering that depression often develops following TBI and greatly disrupts the lives of survivors and their families. In the absence of a solid evidence base for any specific drug, tentative trials of anti-depressant medication weighing vulnerability to risk factors seem appropriate (Hicks et al., 2023).

Several studies agree that some serotonin (citalopram or sertraline) or norepinephrine and DA (methylphenidate) reuptake inhibitors may be effective in reducing depressive symptoms in patients with TBI (Lee et al., 2005; Pangilinan et al., 2010; Gao C. et al., 2022). In particular, DA-enhancing medications are especially suggested to treat cognitive disorders following a TBI (Writer and Schillerstrom, 2009; Traeger et al., 2020), although drugs such as amantadine, which, among other effects, restores DA levels in the striatum, decreases degeneration and apoptosis of DAergic neurons in the SN, and significantly ameliorates depression-like behavior both in patients and in animal models (Sami and Faruqui, 2015; Tan et al., 2015; Chen et al., 2017b).

It has also been pointed out that some natural extracts, such as 2-Pentadecyl-2-Oxazoline, increase DA levels (Boccella et al., 2020) or sinomenine that acts through the D2R ameliorates behavioral alterations in animal models of TBI (Hong et al., 2022). Likewise, it has been documented that the placebo effect, which represents a powerful and effective treatment for various post-TBI complaints, among other mechanisms of action, increases DA levels (Polich et al., 2018). Alternatively, the beneficial effect of listening to music effects of music lies in its ability to control stress and anxiety in post-TBI patients, which can stimulate DA production in the brain (Basile, 2023). Furthermore, a study reported that TBI induced by the accelerated impact model in rats produced depression-like behavior 14 days after trauma. These mood and anxiety disorders were reversed by the administration of bupropion (DA reuptake inhibitor), suggesting that they were closely related to dopaminergic neurotransmission (Mahesh et al., 2010).

On the other hand, it has been shown that the administration of nomifensine maleate (DA reuptake inhibitor) and quinpirole (D2R agonist) decreased the immobility time in the forced swim test, which suggests that drugs enhancing dopaminergic transmission function as antidepressant therapy in rats (Basso et al., 2005).

### 8.4 Insights from epigenetic studies

Furthermore, it is worth remembering that the secondary damage after TBI includes epigenetic and/or genetic expression changes (Chen et al., 2018). Although several authors point out that the study of genes that influence outcomes following TBI is still in its early stages, various genetic polymorphisms associated with the pathophysiology and outcome following TBI have been identified (McAllister, 2009; Bennett et al., 2016; Chen et al., 2018). For example, it has been documented that the apolipoprotein E genotype and TBI have combined effects, resulting in dementia later in life (Puccio and Alexander, 2015). More recently, considerable attention has focused on genes associated with mild and repetitive TBIs, particularly among combat veterans and professional athletes (Fesharaki-Zadeh, 2019). Although suggesting that a “good” or “bad” allele of a specific gene may predispose an individual to better or worse outcomes following injury, it is becoming increasingly apparent that recovery from TBI is polygenic, involving the interaction of numerous genes from multiple pathways (McAllister, 2015).

In particular, about the polymorphism in genes related to the dopaminergic system, those that code for COMT, D2R, ankyrin repeat, and kinase domain containing 1 (ANKK1) and DA transport (SLC6A3) or VMAT have been analyzed in TBI outcomes context (Jordan, 2007). It has been described that the polymorphism of some of them may influence cognitive deficits following TBI. However, their involvement in changes in mood disorders following a TBI needs to be more documented. Only one preliminary report suggests that combining certain COMT and ANKK1 alleles may contribute to worse behavioral performance than the carriage of either risk allele alone (Myrga et al., 2016).

## 9 Discussion

TBI represents a significant public health problem due to high mortality rates and the development of neurological and psychiatric sequelae, including mood, affective, and anxiety disorders that often cause long-lasting disability. It is even pointed out that there is an association between the severity of TBI and cognitive disorders (Shuanglong et al., 2024). The studies shown in this review emphasize that TBI causes alterations in the dopaminergic system, which are associated with mood disorders, including depression and anxiety disorders. For example, after TBI, alterations in the levels of dopamine, its receptors, DAT, and the enzymes involved in its metabolism have been observed, as well as structural and functional changes in the neuronal circuits that connect brain areas specialized in emotional processing with significant dopaminergic innervation and dopaminergic receptors presence, such as the prefrontal cortex, basal ganglia (SN), amygdala and VTA, which could contribute to the development of post-traumatic depression.

It should not be overlooked that the body’s response to TBI is dynamic and can cause immediate or slowly progressive injuries, regardless of severity. The above is relevant because it implies that the appearance of affective or mood disorders could become established gradually as the pathophysiological processes are established in the neuronal circuits involved.

After a TBI, a functional deficit of the monoaminergic circuits occurs, highlighting the dopaminergic system. This deficit varies depending on the pathways or brain regions affected and with time post-TBI. The pathophysiological processes secondary to TBI are associated with chronic neuroinflammation, the generation of oxidative stress, alterations in metabolism, and the destruction (immediate or progressive) of the regions that synthesize dopamine, leading the subject to a hypodopaminergic state, characterized by alterations in DA and DAT and TH concentrations and changes in receptor expression; which together could be a protection mechanism; by increasing circulating levels of dopamine and reducing reuptake systems (Jahan and Tanev, 2023). Each of the dopaminergic pathways presents a different susceptibility to the pathological processes associated with TBI and, therefore, could explain the wide range of clinical signs and symptoms in affected patients, among which are working memory deficits, depression, anxiety, addictions, and behavioral or personality disorders.

Alterations in dopaminergic pathways, caused directly as a response to primary brain injury may participate in developing the neuropsychiatric problems addressed here. However, these alterations can also derive from the progression of the secondary lesion, initiating a positive feedback cycle that will determine the patient’s clinical evolution. As an example of the above, after a TBI, there is an uncontrolled release of ions and neurotransmitters that can cause edema and transient cell membrane depolarization. Uncontrolled transmission leads to a metabolic crisis, where there is mitochondrial damage, decreased ATP production, induction of oxidative stress, increase in reactive oxygen species, and the generation of nitric oxide, patterns that have been observed in both depression and in TBI (Hong et al., 2022). These facilitate neuroinflammation, alter cellular activity, and can damage macromolecules, such as DNA, proteins, and lipids. Elevated levels of pro-inflammatory cytokine have been observed in patients with TBI and depression (Bodnar et al., 2018). The same inflammatory process causes deregulation of the endocrine system, such as the alterations observed in the HPA axis (Luo et al., 2015) and in GH levels (Pavlovic et al., 2019), aggravating metabolic crises. Damage and repair mechanisms, including changes in gene expression, induction of glial inflammatory responses, structural remodeling of proteins, cells, and circuits, and proliferation of neurons and glia, play a decisive role in developing neurobehavioral disorders, including mood disorders. Disruption of cellular processes and persistent low-grade neuroinflammation may predispose some people to depression after TBI (Jahan and Tanev, 2023).

It would be very simplistic to attribute that the depression produced after a TBI is due solely to alterations in the dopaminergic system and not to a multifactorial phenomenon. Various studies indicate that mood disorders are associated with milder levels of TBI (García-Guerrero and Pérez-Moreno, 2014; Uiterwijk et al., 2022), which is very difficult to attribute exclusively to damage to the dopaminergic system. Although damage to this system caused by TBI is an important factor in mood alterations, it must be considered that they have multiple origins, such as an imbalance in aminergic neurotransmission, especially serotonergic neurotransmission, neuroinflammation, and neuroendocrine dysregulation. of the hypothalamic–pituitary–adrenal axis.

An aspect worth highlighting is that there are many more reports of male patients in the clinical studies referred to here. Perhaps this is not surprising given that there is a higher proportion of men who suffer from TBI. However, it has been reported that compared to males, female patients have poorer cognitive recovery and more severe symptoms of depression and anxiety after a TBI (Cnossen et al., 2017; Liu et al., 2024). Likewise, in studies in animal models, those carried out only in males largely predominate.

## 10 Conclusion

Studies discussed in this chapter, in human subjects, as in animal models, provide evidence of a relationship between TBI and mood, affective, and anxiety disorders associated with lesion location. However, it is worth highlighting that although numerous studies, both clinical trials and animal models, show alterations in the dopaminergic system after a TBI, only some of them include tests of mood or affective state, and there are practically no reports that correlate the magnitude or intensity of these two types of variables. Likewise, more studies on animal models need to be conducted, including both males and females.

Nevertheless, these data reinforce the importance of continuing to study the modifications of dopaminergic transmission after a traumatic brain injury and its relationship with psychiatric disorders.

## Author contributions

AM-B: Conceptualization, Writing – original draft, Investigation. RT-C: Investigation, Writing – original draft, Formal analysis, Visualization. MM-V: Investigation, Writing – review & editing, Data curation. AP-A: Data curation, Investigation, Writing – review & editing. MLÁM-C: Writing – review & editing, Resources, Supervision, Validation. AD-R: Project administration, Writing – review & editing. CR: Writing – review & editing, Supervision, Validation. LN: Validation, Writing – review & editing, Conceptualization, Funding acquisition, Writing – original draft.

## References

[ref1] Abdullah J. M. Jaafar H. Muthuraju S. Taha S. Pati S. Rafique M. (2013). Normabaric hyperoxia treatment improved locomotor activity of C57BL/6J mice through enhancing dopamine genes following fluid-percussion injury in striatum. Int. J. Biomed. Sci. 9, 194–204. doi: 10.59566/IJBS.2013.9194, PMID: 24711754 PMC3884788

[ref2] Abdullah M. F. I. L. B. Ng Y. P. Sidi H. B. (2018). Depression and anxiety among traumatic brain injury patients in Malaysia. Asian J. Psychiatr. 37, 67–70. doi: 10.1016/j.ajp.2018.08.017, PMID: 30144779

[ref3] Acosta S. A. Tajiri N. de la Pena I. Bastawrous M. Sanberg P. R. Kaneko Y.. (2015). Alpha-synuclein as a pathological link between chronic traumatic brain injury and Parkinson’s disease. J. Cell. Physiol. 230, 1024–1032. doi: 10.1002/jcp.24830, PMID: 25251017 PMC4328145

[ref4] Agrawal S. Branco R. G. (2016). Neuroprotective measures in children with traumatic brain injury. World J. Crit. Care Med. 5, 36–46. doi: 10.5492/wjccm.v5.i1.36 26855892 PMC4733454

[ref5] Albicini M. Eggleston M. McKinlay A. (2020). The prevalence of traumatic brain injury, comorbid anxiety and other psychiatric disorders in an outpatient child and adolescent mental health service. J. Ment. Health 29, 439–445. doi: 10.1080/09638237.2017.1385733, PMID: 28980490

[ref6] Albicini M. McKinlay A. (2018). Anxiety disorders in adults with childhood traumatic brain injury: evidence of difficulties more than 10 years Postinjury. J. Head Trauma Rehabil. 33, 191–199. doi: 10.1097/HTR.0000000000000312 28520662

[ref7] AlbrechtJ. S.AbarigaS. A.RaoV.WickwireE. M. (2020). Incidence of new neuropsychiatric disorder diagnoses following traumatic brain injury. J. Head Trauma Rehabil. 35, E352–E360. doi: 10.1097/HTR.000000000000055131996603

[ref8] Albrecht J. S. Peters M. E. Smith G. S. Rao V. (2017). Anxiety and post-traumatic stress disorder among Medicare beneficiaries after traumatic brain injury. J. Head Trauma Rehabil. 32, 178–184. doi: 10.1097/HTR.0000000000000266, PMID: 28476057 PMC6935533

[ref9] Almeida-Suhett C. P. Prager E. M. Pidoplichko V. Figueiredo T. H. Marini A. M. Li Z.. (2014). Reduced GABAergic inhibition in the basolateral amygdala and the development of anxiety-like behaviors after mild traumatic brain injury. PLoS One 9:e102627. doi: 10.1371/journal.pone.0102627 25047645 PMC4105413

[ref10] Ayano G. (2016). Dopamine: receptors, functions, synthesis, pathways, locations and mental disorders: review of literatures. J. Ment. Disord. Treat. 2, 1–4. doi: 10.4172/2471-271X.1000120

[ref11] Azouvi P. Arnould A. Dromer E. Vallat-Azouvi C. (2017). Neuropsychology of traumatic brain injury: an expert overview. Rev. Neurol. (Paris) 173, 461–472. doi: 10.1016/j.neurol.2017.07.006 28847474

[ref12] Bales J. W. Kline A. E. Wagner A. K. Dixon C. E. (2010). Targeting dopamine in acute traumatic brain injury. Open Drug Discov. J. 2, 119–128. doi: 10.2174/1877381801002010119, PMID: 22308176 PMC3269831

[ref13] Bales J. W. Wagner A. K. Kline A. E. Dixon C. E. (2009). Persistent cognitive dysfunction after traumatic brain injury: a dopamine hypothesis. Neurosci. Biobehav. Rev. 33, 981–1003. doi: 10.1016/j.neubiorev.2009.03.011, PMID: 19580914 PMC2806224

[ref14] Basile G. (2023). Beneficial effects of music in the healing process of traumatic injuries: perceptual control of suffering and possible abatement of disability conditions. Clin. Ter. 174, 531–536. doi: 10.7417/CT.2023.5021, PMID: 38048117

[ref15] Basso A. M. Gallagher K. B. Bratcher N. A. Brioni J. D. Moreland R. B. Hsieh G. C.. (2005). Antidepressant-like effect of D(2/3) receptor-, but not D(4) receptor-activation in the rat forced swim test. Neuropsychopharmacology 30, 1257–1268. doi: 10.1038/sj.npp.1300677 15688083

[ref16] Baykara B. Aksu I. Buyuk E. Kiray M. Sisman A. R. Baykara B.. (2013). Progesterone treatment decreases traumatic brain injury induced anxiety and is correlated with increased serum IGF-1 levels; prefrontal cortex, amygdala, and hippocampus neuron density; and reduced serum corticosterone levels in immature rats. Biotech. Histochem. 88, 250–257. doi: 10.3109/10520295.2013.769630 23480228

[ref17] Baykara B. Cetin F. Baykara B. Aksu I. Dayi A. Kiray M.. (2012). Anxiety caused by traumatic brain injury correlates to decreased prefrontal cortex VEGF immunoreactivity and neuron density in immature rats. Turk. Neurosurg. 22, 604–610. doi: 10.5137/1019-5149.JTN.5633-11.1, PMID: 23015338

[ref18] Beaulieu J. M. Gainetdinov R. R. (2011). The physiology, signaling, and pharmacology of dopamine receptors. Pharmacol. Rev. 63, 182–217. doi: 10.1124/pr.110.002642 21303898

[ref19] Beitchman J. A. Griffiths D. R. Hur Y. Ogle S. B. Bromberg C. E. Morrison H. W.. (2020). Experimental traumatic brain injury induces chronic glutamatergic dysfunction in amygdala circuitry known to regulate anxiety-like behavior. Front. Neurosci. 13:1434. doi: 10.3389/fnins.2019.01434, PMID: 32038140 PMC6985437

[ref20] Bennett E. R. Reuter-Rice K. Laskowitz D. T. (2016). Genetic influences in traumatic brain injury. En Laskowitz D. Grant G. (Eds.), Translational research in traumatic brain injury (Capítulo 9). Boca Raton (FL): CRC Press/Taylor and Francis Group. 26583176

[ref21] Bhowmick S. Malat A. Caruso D. Ponery N. D'Mello V. Finn C.. (2021). Intercellular adhesion Molecule-1-induced posttraumatic brain injury neuropathology in the prefrontal cortex and Hippocampus leads to sensorimotor function deficits and psychological stress. eNeuro 8, ENEURO.0242–ENEU21.2021. doi: 10.1523/ENEURO.0242-21.2021, PMID: 34135004 PMC8287878

[ref22] Björklund A. Dunnett S. B. (2007). Dopamine neuron systems in the brain: an update. Trends Neurosci. 30, 194–202. doi: 10.1016/j.tins.2007.03.006, PMID: 17408759

[ref23] Boccella S. Iannotta M. Cristiano C. Iannotti F. A. Bello F. D. Guida F.. (2020). Treatment with 2-Pentadecyl-2-Oxazoline restores mild traumatic brain injury-induced sensorial and neuropsychiatric dysfunctions. Front. Pharmacol. 11:91. doi: 10.3389/fphar.2020.00091 32161542 PMC7052365

[ref24] Bodnar C. N. Morganti J. M. Bachstetter A. D. (2018). Depression following a traumatic brain injury: uncovering cytokine dysregulation as a pathogenic mechanism. Neural Regen. Res. 13, 1693–1704. doi: 10.4103/1673-5374.238604 30136679 PMC6128046

[ref25] Braga M. F. M. Juranek J. Eiden L. E. Li Z. Figueiredo T. H. de Araujo Furtado M.. (2022). GABAergic circuits of the basolateral amygdala and generation of anxiety after traumatic brain injury. Amino Acids 54, 1229–1249. doi: 10.1007/s00726-022-03184-y, PMID: 35798984

[ref26] Broussard J. I. Acion L. De Jesús-Cortés H. Yin T. Britt J. K. Salas R.. (2018). Repeated mild traumatic brain injury produces neuroinflammation, anxiety-like behavior and impaired spatial memory in mice. Brain Inj. 32, 113–122. doi: 10.1080/02699052.2017.1380228, PMID: 29156991

[ref27] Bryant R. A. O'Donnell M. L. Creamer M. McFarlane A. C. Clark C. R. Silove D. (2010). The psychiatric sequelae of traumatic injury. Am. J. Psychiatry 167, 312–320. doi: 10.1176/appi.ajp.2009.09050617 20048022

[ref28] Capizzi A. Woo J. Verduzco-Gutierrez M. (2020). Traumatic brain injury: an overview of epidemiology, pathophysiology, and medical management. Med. Clin. North Am. 104, 213–238. doi: 10.1016/j.mcna.2019.11.001 32035565

[ref29] Carlson S. W. Dixon C. E. (2018). Lithium improves dopamine neurotransmission and increases dopaminergic protein abundance in the striatum after traumatic brain injury. J. Neurotrauma 35, 2827–2836. doi: 10.1089/neu.2017.5509, PMID: 29699444 PMC6247981

[ref30] Chen Y. H. Huang E. Y. Kuo T. T. Hoffer B. J. Miller J. Chou Y. C.. (2017a). Dopamine release in the nucleus accumbens is altered following traumatic brain injury. Neuroscience 348, 180–190. doi: 10.1016/j.neuroscience.2017.02.001 28196657

[ref31] Chen Y. H. Huang E. Y. Kuo T. T. Ma H. I. Hoffer B. J. Tsui P. F.. (2015). Dopamine release impairment in striatum after different levels of cerebral cortical fluid percussion injury. Cell Transplant. 24, 2113–2128. doi: 10.3727/096368914X683584, PMID: 25198499

[ref32] Chen Y. H. Huang E. Y. Kuo T. T. Miller J. Chiang Y. H. Hoffer B. J. (2017b). Impact of traumatic brain injury on dopaminergic transmission. Cell Transplant. 26, 1156–1168. doi: 10.1177/0963689717714105, PMID: 28933212 PMC5657731

[ref33] Chen Y. H. Kuo T. T. Huang E. Y. K. Hoffer B. J. Kao J. H. Chou Y. C.. (2018). Nicotine-induced conditional place preference is affected by head injury: correlation with dopamine release in the nucleus accumbens shell. Int. J. Neuropsychopharmacol. 21, 949–961. doi: 10.1093/ijnp/pyy055, PMID: 29905798 PMC6165954

[ref34] Cnossen M. C. Scholten A. C. Lingsma H. F. Synnot A. Haagsma J. Steyerberg P. E. W.. (2017). Predictors of major depression and posttraumatic stress disorder following traumatic brain injury: a systematic review and Meta-analysis. J. Neuropsychiatr. Clin. Neurosci. 29, 206–224. doi: 10.1176/appi.neuropsych.16090165 28193126

[ref35] Corrigan F. Arulsamy A. Shultz S. R. Wright D. K. Collins-Praino L. E. (2023). Initial severity of injury has little effect on the temporal profile of long-term deficits in locomotion, anxiety, and cognitive function after diffuse traumatic brain injury. Neurotrauma Rep. 4, 41–50. doi: 10.1089/neur.2022.0057, PMID: 36726871 PMC9886190

[ref9001] Corrigan J. D. Selassie A. W. Orman J. A. (2010). The epidemiology of traumatic brain injury. J. Head Trauma Rehabil. 25, 72–80. doi: 10.1097/HTR.0b013e3181ccc8b4 20234226

[ref36] Cristofori I. Levin H. S. (2015). Traumatic brain injury and cognition. Handb. Clin. Neurol. 128, 579–611. doi: 10.1016/B978-0-444-63521-1.00037-6 25701909

[ref37] D'Amico R. Trovato Salinaro A. Fusco R. Cordaro M. Impellizzeri D. Scuto M.. (2021). Hericium erinaceus and Coriolus versicolor modulate molecular and biochemical changes after traumatic brain injury. Antioxidants (Basel) 10:898. doi: 10.3390/antiox10060898, PMID: 34199629 PMC8228340

[ref38] Dewan M. C. Rattani A. Gupta S. Baticulon R. E. Hung Y. C. Punchak M.. (2018). Estimating the global incidence of traumatic brain injury. J. Neurosurg. 130, 1080–1097. doi: 10.3171/2017.10.JNS17352 29701556

[ref39] Dixon K. J. (2017). Pathophysiology of traumatic brain injury. Phys. Med. Rehabil. Clin. N. Am. 28, 215–225. doi: 10.1016/j.pmr.2016.12.001 28390509

[ref40] Donnemiller E. Brenneis C. Wissel J. Scherfler C. Poewe W. Riccabona G.. (2000). Impaired dopaminergic neurotransmission in patients with traumatic brain injury: a SPECT study using 123I-beta-CIT and 123I-IBZM. Eur. J. Nucl. Med. 27, 1410–1414. doi: 10.1007/s002590000308, PMID: 11007526

[ref41] Dunlop B. W. Nemeroff C. B. (2007). The role of dopamine in the pathophysiology of depression. Arch. Gen. Psychiatry 64, 327–337. doi: 10.1001/archpsyc.64.3.327 17339521

[ref42] Edut S. Rubovitch V. Rehavi M. Schreiber S. Pick C. G. (2014). A study on the mechanism by which MDMA protects against dopaminergic dysfunction after minimal traumatic brain injury (mTBI) in mice. J. Mol. Neurosci. 54, 684–697. doi: 10.1007/s12031-014-0399-z, PMID: 25124230

[ref43] Edvinsson L. Owman C. Rosengren E. West K. A. (1971). Brain concentrations of dopamine, noradrenaline, 5-hydroxytryptamine, and homovanillic acid during intracranial hypertension following traumatic brain injury in rabbit. Acta Neurol. Scand. 47, 458–463. doi: 10.1111/j.1600-0404.1971.tb07500.x, PMID: 5123487

[ref44] Eliasen M. H. Petersen J. Benros M. E. Osler M. (2021). Number of traumatic brain injuries and temporal associations with depression: a register-based cohort study. Acta Psychiatr. Scand. 144, 407–414. doi: 10.1111/acps.13347, PMID: 34231201

[ref45] Feldman Z. Contant C. F. Pahwa R. Goodman J. C. Robertson C. S. Narayan R. K.. (1993). The relationship between hormonal mediators and systemic hypermetabolism after severe head injury. J. Trauma 34, 806–812. doi: 10.1097/00005373-199306000-00010, PMID: 8315675

[ref46] Fesharaki-Zadeh A. (2019). Chronic traumatic encephalopathy: a brief overview. Front. Neurol. 10:713. doi: 10.3389/fneur.2019.00713, PMID: 31333567 PMC6616127

[ref47] Fleminger S. Ponsford J. (2005). Long term outcome after traumatic brain injury. BMJ 331, 1419–1420. doi: 10.1136/bmj.331.7530.1419 16356951 PMC1315633

[ref48] Fromm L. Heath D. L. Vink R. Nimmo A. J. (2004). Magnesium attenuates post-traumatic depression/anxiety following diffuse traumatic brain injury in rats. J. Am. Coll. Nutr. 23, 529S–533S. doi: 10.1080/07315724.2004.10719396, PMID: 15466958

[ref49] Gao C. Nie M. Huang J. Tian Y. Wang D. Zhang J.. (2022). Pharmacotherapy for mild traumatic brain injury: an overview of the current treatment options. Expert. Opin. Pharmacother. 23, 805–813. doi: 10.1080/14656566.2022.2054328, PMID: 35290753

[ref50] Gao L. Xue Q. Gong S. Li G. Tong W. Fan M.. (2022). Structural and functional alterations of substantia nigra and associations with anxiety and depressive symptoms following traumatic brain injury. Front. Neurol. 13:719778. doi: 10.3389/fneur.2022.719778, PMID: 35449518 PMC9017679

[ref51] García-Guerrero C. E. Pérez-Moreno B. (2014). Risk factors related to the development of depression after traumatic brain injury. Neuropsicol. Latinoam. 6, 25–46. doi: 10.5579/rnl.2014.0155

[ref52] Grace A. A. Rosenkranz J. A. (2002). Regulation of conditioned responses of basolateral amygdala neurons. Physiol. Behav. 77, 489–493. doi: 10.1016/S0031-9384(02)00909-5 12526988

[ref53] Green R. E. Colella B. Christensen B. Johns K. Frasca D. Bayley M.. (2008). Examining moderators of cognitive recovery trajectories after moderate to severe traumatic brain injury. Arch. Phys. Med. Rehabil. 89, S16–S24. doi: 10.1016/j.apmr.2008.09.551 19081437

[ref54] Guillamondegui O. D. Montgomery S. A. Phibbs F. T. McPheeters M. L. Alexander P. T. Jerome R. N.. (2011). Traumatic brain injury and depression. Rockville (MD): Agency for Healthcare Research and Quality (US). 21938798

[ref55] Hamill R. W. Woolf P. D. McDonald J. V. Lee L. A. Kelly M. (1987). Catecholamines predict outcome in traumatic brain injury. Ann. Neurol. 21, 438–443. doi: 10.1002/ana.410210504, PMID: 3592639

[ref56] Harsing L. G. (2008). Dopamine and the Dopaminergic Systems of the Brain. In: Handbook of Neurochemistry and Molecular Neurobiology. eds Lajtha A. Vizi E. S. (Boston, MA: Springer).

[ref57] Hasler G. Fromm S. Carlson P. J. Luckenbaugh D. A. Waldeck T. Geraci M.. (2008). Neural response to catecholamine depletion in Unmedicated subjects with major depressive disorder in remission and healthy subjects. Arch. Gen. Psychiatry 65, 521–531. doi: 10.1001/archpsyc.65.5.521, PMID: 18458204 PMC2676777

[ref58] Hercher C. Turecki G. Mechawar N. (2009). Through the looking glass: examining neuroanatomical evidence for cellular alterations in major depression. J. Psychiatr. Res. 43, 947–961. doi: 10.1016/j.jpsychires.2009.01.006, PMID: 19233384

[ref59] Heshmati M. Russo S. J. (2015). Anhedonia and the brain reward circuitry in depression. Curr. Behav. Neurosci. Rep. 2, 146–153. doi: 10.1007/s40473-015-0044-3, PMID: 26525751 PMC4626008

[ref60] Hicks A. J. Clay F. J. James A. C. Hopwood M. Ponsford J. L. (2023). Effectiveness of pharmacotherapy for depression after adult traumatic brain injury: an umbrella review. Neuropsychol. Rev. 33, 393–431. doi: 10.1007/s11065-022-09543-6, PMID: 35699850 PMC10148771

[ref61] Hong H. Lu X. Lu Q. Huang C. Cui Z. (2022). Potential therapeutic effects and pharmacological evidence of sinomenine in central nervous system disorders. Front. Pharmacol. 13:1015035. doi: 10.3389/fphar.2022.1015035, PMID: 36188580 PMC9523510

[ref62] Hu E. Tang T. Li Y. M. Li T. Zhu L. Ding R. Q.. (2023). Spatial amine metabolomics and histopathology reveal localized brain alterations in subacute traumatic brain injury and the underlying mechanism of herbal treatment. CNS Neurosci. Ther. 30:e14231. doi: 10.1111/cns.14231, PMID: 37183394 PMC10915989

[ref63] Huang M. Lewine J. D. Lee R. R. (2020). Magnetoencephalography for mild traumatic brain injury and posttraumatic stress disorder. Neuroimaging Clin. N. Am. 30, 175–192. doi: 10.1016/j.nic.2020.02.003, PMID: 32336405

[ref64] Huang E. Y. K. Tsui P. F. Kuo T. T. Tsai J. Chou Y. C. Ma H. I.. (2014). Amantadine ameliorates dopamine-releasing deficits and behavioral deficits in rats after fluid percussion injury. PLoS One 9:e86354. doi: 10.1371/journal.pone.0086354, PMID: 24497943 PMC3907421

[ref65] Hutson C. B. Lazo C. R. Mortazavi F. Giza C. C. Hovda D. Chesselet M. F. (2011). Traumatic brain injury in adult rats causes progressive nigrostriatal dopaminergic cell loss and enhanced vulnerability to the pesticide paraquat. J. Neurotrauma 28, 1783–1801. doi: 10.1089/neu.2010.1723, PMID: 21644813 PMC3172882

[ref66] Impellizzeri D. Campolo M. Bruschetta G. Crupi R. Cordaro M. Paterniti I.. (2016). Traumatic brain injury leads to development of Parkinson’s disease related pathology in mice. Front. Neurosci. 10:458. doi: 10.3389/fnins.2016.00458, PMID: 27790086 PMC5061819

[ref67] Jahan A. B. Tanev K. (2023). Neurobiological mechanisms of depression following traumatic brain injury. Brain Inj. 37, 24–33. doi: 10.1080/02699052.2022.2145362 36373974

[ref68] Jenkins P. O. Mehta M. A. Sharp D. J. (2016). Catecholamines and cognition after traumatic brain injury. Brain 139, 2345–2371. doi: 10.1093/brain/aww128, PMID: 27256296 PMC4995357

[ref69] Jenkins P. O. Roussakis A. A. De Simoni S. Bourke N. Fleminger J. Cole J.. (2020). Distinct dopaminergic abnormalities in traumatic brain injury and Parkinson’s disease. J. Neurol. Neurosurg. Psychiatry 91, 631–637. doi: 10.1136/jnnp-2019-321759, PMID: 32381639

[ref70] Johnson D. Roethig-Johnston K. Richards D. (1993). Biochemical parameters of recovery in acute severe head injury. Br. J. Neurosurg. 7, 53–59. doi: 10.3109/02688699308995056, PMID: 8094621

[ref71] Jolly A. E. Raymont V. Cole J. H. Whittington A. Scott G. De Simoni S.. (2019). Dopamine D2/D3 receptor abnormalities after traumatic brain injury and their relationship to post-traumatic depression. NeuroImage Clin. 24:101950. doi: 10.1016/j.nicl.2019.101950, PMID: 31352218 PMC6664227

[ref72] Jones N. C. Cardamone L. Williams J. P. Salzberg M. R. Myers D. O’Brien T. J. (2008). Experimental traumatic brain injury induces a pervasive hyperanxious phenotype in rats. J. Neurotrauma 25, 1367–1374. doi: 10.1089/neu.2008.0641, PMID: 19061380

[ref73] Jordan B. D. (2007). Genetic influences on outcome following traumatic brain injury. Neurochem. Res. 32, 905–915. doi: 10.1007/s11064-006-9251-3 17342413

[ref74] Jorge R. E. (2015). Mood disorders. Handb. Clin. Neurol. 128, 613–631. doi: 10.1016/B978-0-444-63521-1.00038-8 25701910

[ref75] Juengst S. B. Kumar R. G. Wagner A. K. (2017). A narrative literature review of depression following traumatic brain injury: prevalence, impact, and management challenges. Psychol. Res. Behav. Manag. 10, 175–186. doi: 10.2147/PRBM.S113264, PMID: 28652833 PMC5476717

[ref76] Karelina K. Gaier K. R. Weil Z. M. (2017). Traumatic brain injuries during development disrupt dopaminergic signaling. Exp. Neurol. 297, 110–117. doi: 10.1016/j.expneurol.2017.08.003 28802560 PMC6033277

[ref77] Kaukas L. Holmes J. L. Rahimi F. Collins-Praino L. Corrigan F. (2021). Injury during adolescence leads to sex-specific executive function deficits in adulthood in a pre-clinical model of mild traumatic brain injury. Behav. Brain Res. 402:113067. doi: 10.1016/j.bbr.2020.113067, PMID: 33333110

[ref78] Khellaf A. Khan D. Z. Helmy A. (2019). Recent advances in traumatic brain injury. J. Neurol. 266, 2878–2889. doi: 10.1007/s00415-019-09541-4, PMID: 31563989 PMC6803592

[ref79] Klein M. O. Battagello D. S. Cardoso A. R. Hauser D. N. Bittencourt J. C. Correa R. G. (2019). Dopamine: functions, signaling, and association with neurological diseases. Cell. Mol. Neurobiol. 39, 31–59. doi: 10.1007/s10571-018-0632-3 30446950 PMC11469830

[ref80] Ko I. G. Kim C. J. Kim H. (2019). Treadmill exercise improves memory by up-regulating dopamine and down-regulating D2 dopamine receptor in traumatic brain injury rats. J. Exerc. Rehabil. 15, 504–511. doi: 10.12965/jer.1938316.158 31523669 PMC6732546

[ref81] Ko J. H. Strafella A. P. (2012). Dopaminergic neurotransmission in the human brain: new lessons from perturbation and imaging. Neuroscientist 18, 149–168. doi: 10.1177/1073858411401413, PMID: 21536838 PMC3479149

[ref82] Kobori N. Clifton G. L. Dash P. K. (2006). Enhanced catecholamine synthesis in the prefrontal cortex after traumatic brain injury: implications for prefrontal dysfunction. J. Neurotrauma 23, 1094–1102. doi: 10.1089/neu.2006.23.1094, PMID: 16866622

[ref83] Koenigs M. Grafman J. (2009). The functional neuroanatomy of depression: distinct roles for ventromedial and dorsolateral prefrontal cortex. Behav. Brain Res. 201, 239–243. doi: 10.1016/j.bbr.2009.03.004, PMID: 19428640 PMC2680780

[ref84] Kreitzer N. Ancona R. Mccullumsmith C. Kurowski B. G. Foreman B. Ngwenya L. B.. (2019). The effect of antidepressants on depression after traumatic brain injury: a meta-analysis. J. Head Trauma Rehabil. 34, E47–E54. doi: 10.1097/HTR.0000000000000439, PMID: 30169440 PMC8730802

[ref85] Kumar S. Singh G. (2023). Pharmacological potential of zonisamide and *Nigella sativa* per se and combination in high-impact trauma device-induced traumatic brain injury in *Drosophila melanogaster*. Fundam. Clin. Pharmacol. 37, 577–588. doi: 10.1111/fcp.12857, PMID: 36424858

[ref86] Laliberté Durish C. Pereverseff R. S. Yeates K. O. (2018). Depression and depressive symptoms in pediatric traumatic brain injury: a scoping review. J. Head Trauma Rehabil. 33, E18–E30. doi: 10.1097/HTR.0000000000000343, PMID: 28926485 PMC5857396

[ref87] Lamontagne G. Belleville G. Beaulieu-Bonneau S. Souesme G. Savard J. Sirois M. J.. (2022). Anxiety symptoms and disorders in the first year after sustaining mild traumatic brain injury. Rehabil. Psychol. 67, 90–99. doi: 10.1037/rep0000422 34843337

[ref88] Lan Y. L. Li S. Lou J. C. Ma X. C. Zhang B. (2019). The potential roles of dopamine in traumatic brain injury: a preclinical and clinical update. Am. J. Transl. Res. 11, 2616–2631, PMID: 31217842 PMC6556629

[ref89] Leconte C. Benedetto C. Lentini F. Simon K. Ouaazizi C. Taib T.. (2020). Histological and behavioral evaluation after traumatic brain injury in mice: a ten months follow-up study. J. Neurotrauma 37, 1342–1357. doi: 10.1089/neu.2019.6679, PMID: 31830858

[ref90] Lee H. Kim S. W. Kim J. M. Shin I. S. Yang S. J. Yoon J. S. (2005). Comparing effects of methylphenidate, sertraline and placebo on neuropsychiatric sequelae in patients with traumatic brain injury. Hum. Psychopharmacol. 20, 97–104. doi: 10.1002/hup.668 15641125

[ref110] Leker R. R. Shohami E. (2002). Cerebral ischemia and trauma-different etiologies yet similar mechanisms: neuroprotective opportunities. Brain Res. Rev. 39, 55–73. doi: 10.1016/s0165-0173(02)00157-1 12086708

[ref91] Liu M. Bachstetter A. D. Cass W. A. Lifshitz J. Bing G. (2017). Pioglitazone attenuates neuroinflammation and promotes dopaminergic neuronal survival in the nigrostriatal system of rats after diffuse brain injury. J. Neurotrauma 34, 414–422. doi: 10.1089/neu.2015.4361 27142118

[ref92] Liu T. Yu S. Liu M. Zhao Z. Yuan J. Sha Z.. (2024). Cognitive impairment in Chinese traumatic brain injury patients: from challenge to future perspectives. Front. Neurosci. 18:1361832. doi: 10.3389/fnins.2024.1361832 38529265 PMC10961372

[ref93] Lowing J. L. Susick L. L. Caruso J. P. Provenzano A. M. Raghupathi R. Conti A. C. (2014). Experimental traumatic brain injury alters ethanol consumption and sensitivity. J. Neurotrauma 31, 1700–1710. doi: 10.1089/neu.2013.3286, PMID: 24934382 PMC4180526

[ref94] Luo L. Chai Y. Jiang R. Chen X. Yan T. (2015). Cortisol supplement combined with psychotherapy and citalopram improves depression outcomes in patients with hypocortisolism after traumatic brain injury. Aging Dis. 6, 418–425. doi: 10.14336/AD.2015.0507, PMID: 26618043 PMC4657813

[ref95] Maas A. I. Stocchetti N. Bullock R. (2008). Moderate and severe traumatic brain injury in adults. Lancet Neurol. 7, 728–741. doi: 10.1016/S1474-4422(08)70164-9 18635021

[ref96] Mahesh R. Pandey D. K. Katiyar S. Kukade G. Viyogi S. Rudra A. (2010). Effect of antidepressants on neuro-behavioral consequences following impact accelerated traumatic brain injury in rats. Indian J. Exp. Biol. 8, 466–473. 20795363

[ref97] Mallya S. Sutherland J. Pongracic S. Mainland B. Ornstein T. J. (2015). The manifestation of anxiety disorders after traumatic brain injury: a review. J. Neurotrauma 32, 411–421. doi: 10.1089/neu.2014.3504, PMID: 25227240

[ref98] Markianos M. Seretis A. Kotsou S. Baltas I. Sacharogiannis H. (1992). CSF neurotransmitter metabolites and short-term outcome of patients in coma after head injury. Acta Neurol. Scand. 86, 190–193. doi: 10.1111/j.1600-0404.1992.tb05064.x, PMID: 1384260

[ref99] Massucci J. L. Kline A. E. Ma X. Zafonte R. D. Dixon C. E. (2004). Time dependent alterations in dopamine tissue levels and metabolism after experimental traumatic brain injury in rats. Neurosci. Lett. 372, 127–131. doi: 10.1016/j.neulet.2004.09.026 15531102

[ref100] McAllister T. W. (2009). Polymorphisms in genes modulating the dopamine system: do they influence outcome and response to medication after traumatic brain injury? J. Head Trauma Rehabil. 24, 65–68. doi: 10.1097/HTR.0b013e3181996e6b, PMID: 19158598 PMC3169998

[ref101] McAllister T. W. (2015). Genetic factors in traumatic brain injury. Handb. Clin. Neurol. 128, 723–739. doi: 10.1016/B978-0-444-63521-1.00045-5 25701917

[ref102] McIntosh T. K. Yu T. Gennarelli T. A. (1994). Alterations in regional brain catecholamine concentrations after experimental brain injury in the rat. J. Neurochem. 63, 1426–1433. doi: 10.1046/j.1471-4159.1994.63041426.x, PMID: 7931293

[ref103] Meiser J. Weindl D. Hiller K. (2013). Complexity of dopamine metabolism. Cell Commun. Signal. 11:34. doi: 10.1186/1478-811X-11-34, PMID: 23683503 PMC3693914

[ref104] Menon K. Schwab K. Wright D. W. Maas A. I. (2010). Demographics and clinical assessment working Group of the International and Interagency Initiative toward common data elements for research on traumatic brain injury and psychological health. Position statement: definition of traumatic brain injury. Arch. Phys. Med. Rehabil. 91, 1637–1640. doi: 10.1016/j.apmr.2010.05.017, PMID: 21044706

[ref105] Mulvihill K. G. (2019). Presynaptic regulation of dopamine release: role of the DAT and VMAT2 transporters. Neurochem. Int. 122, 94–105. doi: 10.1016/j.neuint.2018.11.004 30465801

[ref106] Muthuraju S. Islam M. R. Pati S. Jaafar H. Abdullah J. M. Yusoff K. M. (2014). Normobaric hyperoxia treatment prevents early alteration in dopamine level in mice striatum after fluid percussion injury: a biochemical approach. Int. J. Neurosci. 125, 686–692. doi: 10.3109/00207454.2014.961065 25180987

[ref107] Myöhänen T. T. Schendzielorz N. Männistö P. T. (2010). Distribution of catechol-O-methyltransferase (COMT) proteins and enzymatic activities in wild-type and soluble COMT deficient mice. J. Neurochem. 113, 1632–1643. doi: 10.1111/j.1471-4159.2010.06723.x 20374420

[ref108] Myrga J. M. Juengst S. B. Failla M. D. Conley Y. P. Arenth P. M. Grace A. A.. (2016). COMT and ANKK1 genetics interact with depression to influence behavior following severe TBI: an initial assessment. Neurorehabil. Neural Repair 30, 920–930. doi: 10.1177/1545968316648409, PMID: 27154305 PMC5048493

[ref109] Nestler E. J. Carlezon W. A. (2006). The mesolimbic dopamine reward circuit in depression. Biol. Psychiatry 59, 1151–1159. doi: 10.1016/j.biopsych.2005.09.018 16566899

[ref111] Newberg A. B. Serruya M. Gepty A. Intenzo C. Lewis T. Amen D.. (2014). Clinical comparison of 99mTc exametazime and 123I Ioflupane SPECT in patients with chronic mild traumatic brain injury. PLoS One 9:e87009. doi: 10.1371/journal.pone.0087009, PMID: 24475210 PMC3901727

[ref112] Nieoullon A. (2002). Dopamine and the regulation of cognition and attention. Prog. Neurobiol. 67, 53–83. doi: 10.1016/s0301-0082(02)00011-4, PMID: 12126656

[ref113] Ortiz O. Delgado-García J. M. Espadas I. Bahí A. Trullas R. Dreyer J. L.. (2010). Associative learning and CA3–CA1 synaptic plasticity are impaired in D1R null, Drd1a−/− mice and in hippocampal siRNA silenced Drd1a mice. J. Neurosci. 30, 12288–12300. doi: 10.1523/JNEUROSCI.2655-10.2010, PMID: 20844125 PMC6633447

[ref114] Osborn A. J. Mathias J. L. Fairweather-Schmidt A. K. (2014). Depression following adult, non-penetrating traumatic brain injury: a meta-analysis examining methodological variables and sample characteristics. Neurosci. Biobehav. Rev. 47, 1–15. doi: 10.1016/j.neubiorev.2014.07.007 25038422

[ref115] Osborn A. J. Mathias J. L. Fairweather-Schmidt A. K. (2016). Prevalence of anxiety following adult traumatic brain injury: a meta-analysis comparing measures, samples and postinjury intervals. Neuropsychology 30, 247–261. doi: 10.1037/neu0000221, PMID: 26146855

[ref116] Pangilinan P. H. Giacoletti-Argento A. Shellhaas R. Hurvitz E. A. Hornyak J. E. (2010). Neuropharmacology in pediatric brain injury: a review. PM R 2, 1127–1140. doi: 10.1016/j.pmrj.2010.07.007, PMID: 21145525

[ref117] Pavlovic D. Pekic S. Stojanovic M. Popovic V. (2019). Traumatic brain injury: neuropathological, neurocognitive and neurobehavioral sequelae. Pituitary 22, 270–282. doi: 10.1007/s11102-019-00957-9, PMID: 30929221

[ref118] Perry D. C. Sturm V. E. Peterson M. J. Pieper C. F. Bullock T. Boeve B. F.. (2016). Association of traumatic brain injury with subsequent neurological and psychiatric disease: a meta-analysis. J. Neurosurg. 124, 511–526. doi: 10.3171/2015.2.JNS14503 26315003 PMC4751029

[ref119] Pinto P. S. Meoded A. Poretti A. Tekes A. Huisman T. A. (2012). The unique features of traumatic brain injury in children. Review of the characteristics of the pediatric skull and brain, mechanisms of trauma, patterns of injury, complications, and their imaging findings--part 2. J. Neuroimaging 22, e18–e41. doi: 10.1111/j.1552-6569.2011.00690.x 22303964

[ref120] Pischiutta F. Micotti E. Hay J. R. Marongiu I. Sammali E. Tolomeo D.. (2018). Single severe traumatic brain injury produces progressive pathology with ongoing contralateral white matter damage one year after injury. Exp. Neurol. 300, 167–178. doi: 10.1016/j.expneurol.2017.11.003, PMID: 29126888 PMC5745280

[ref121] Plantier D. Luauté J. SOFMER group (2016). Drugs for behavior disorders after traumatic brain injury: systematic review and expert consensus leading to French recommendations for good practice. Ann. Phys. Rehabil. Med. 59, 42–57. doi: 10.1016/j.rehab.2015.10.003 26797170

[ref122] Polich G. Iaccarino M. A. Kaptchuk T. J. Morales-Quezada L. Zafonte R. (2018). Placebo effects in traumatic brain injury. J. Neurotrauma 35, 1205–1212. doi: 10.1089/neu.2017.5506, PMID: 29343158 PMC6016098

[ref123] Ponsford J. Lee N. K. Wong D. Mckay A. Haines K. Downing M.. (2020). Factors associated with response to adapted cognitive behavioral therapy for anxiety and depression following traumatic brain injury. J. Head Trauma Rehabil. 35, 117–126. doi: 10.1097/HTR.0000000000000510, PMID: 31365437

[ref124] Puccio A. M. Alexander S. (2015). Chapter 4 genomics, transcriptomics, and epigenomics in traumatic brain injury research. Annu. Rev. Nurs. Res. 33, 75–109. doi: 10.1891/0739-6686.33.75, PMID: 25946384

[ref125] Rana A. Singh S. Deshmukh R. Kumar A. (2020). Pharmacological potential of tocopherol and doxycycline against traumatic brain injury-induced cognitive/motor impairment in rats. Brain Inj. 34, 1039–1050. doi: 10.1080/02699052.2020.1772508, PMID: 32493074

[ref126] Robert S. (2020). Traumatic brain injury and mood disorders. Ment.Health Clin. 10, 335–345. doi: 10.9740/mhc.2020.11.335, PMID: 33224691 PMC7653730

[ref127] Rodrigues T. B. Granado N. Ortiz O. Cerdán S. Moratalla R. (2007). Metabolic interactions between glutamatergic and dopaminergic neurotransmitter systems are mediated through D(1) dopamine receptors. J. Neurosci. Res. 85, 3284–3293. doi: 10.1002/jnr.21302, PMID: 17455302

[ref128] Ruttan L. Martin K. Liu A. Colella B. Green R. E. (2008). Long-term cognitive outcome in moderate to severe traumatic brain injury: a meta-analysis examining timed and untimed tests at 1 and 4.5 or more years after injury. Arch. Phys. Med. Rehabil. 89, S69–S76. doi: 10.1016/j.apmr.2008.07.007 19081444

[ref129] Ryttersgaard T. O. Johnsen S. P. Riis J. Mogensen P. H. Bjarkam C. R. (2020). Prevalence of depression after moderate to severe traumatic brain injury among adolescents and young adults: a systematic review. Scand. J. Psychol. 61, 297–306. doi: 10.1111/sjop.12587 31774181

[ref130] Sadock B. J. Sadock V. A. Ruiz P. (2015). “Kaplan and Sadock’s synopsis of psychiatry” in Behavioral sciences/clinical psychiatry. 11th ed (Philadelphia (PA): Wolters Kluwer).

[ref131] Sameh G. Islem F. Samar A. Hedi C. Mounir B. Habib E. M. (2021). Neuropsychological and behavioral disorders, functional outcomes and quality of life in traumatic brain injury victims. Pan Afr. Med. J. 38:346. doi: 10.11604/pamj.2021.38.346.16120, PMID: 34367425 PMC8308941

[ref132] Sami M. B. Faruqui R. (2015). The effectiveness of dopamine agonists for treatment of neuropsychiatric symptoms post brain injury and stroke. Acta Neuropsychiat. 27, 317–326. doi: 10.1017/neu.2015.17, PMID: 25850757

[ref133] Sauerbeck A. Hunter R. Bing G. Sullivan P. G. (2012). Traumatic brain injury and trichloroethylene exposure interact and produce functional, histological, and mitochondrial deficits. Exp. Neurol. 234, 85–94. doi: 10.1016/j.expneurol.2011.12.012, PMID: 22201550 PMC3294257

[ref134] Schindler A. G. Meabon J. S. Pagulayan K. F. Hendrickson R. C. Meeker K. D. Cline M.. (2017). Blast-related disinhibition and risk seeking in mice and combat veterans: potential role for dysfunctional phasic dopamine release. Neurobiol. Dis. 106, 23–34. doi: 10.1016/j.nbd.2017.06.004, PMID: 28619545

[ref135] Schmidt R. H. Grady M. S. (1995). Loss of forebrain cholinergic neurons following fluid-percussion injury: implications for cognitive impairment in closed head injury. J. Neurosurg. 83, 496–502. doi: 10.3171/jns.1995.83.3.0496, PMID: 7666229

[ref136] Schultz W. (1998). Predictive reward signal of dopamine neurons. J. Neurophysiol. 80, 1–27. doi: 10.1152/jn.1998.80.1.1 9658025

[ref137] Sekhon S. Gupta V. (2023). Mood disorder. FL: StatPearls Publishing. 32644337

[ref138] Selvakumar G. P. Ahmed M. E. Iyer S. S. Thangavel R. Kempuraj D. Raikwar S. P.. (2020). Absence of glia maturation factor protects from axonal injury and motor behavioral impairments after traumatic brain injury. Exp. Neurobiol. 29, 230–248. doi: 10.5607/en20017, PMID: 32565489 PMC7344375

[ref139] Shen H. Harvey B. K. Chiang Y. H. Pick C. G. Wang Y. (2011). Methamphetamine potentiates behavioral and electrochemical responses after mild traumatic brain injury in mice. Brain Res. 1368, 248–253. doi: 10.1016/j.brainres.2010.10.014, PMID: 20950593 PMC3014394

[ref140] Shimada R. Abe K. Furutani R. Kibayashi K. (2014). Changes in dopamine transporter expression in the midbrain following traumatic brain injury: an immunohistochemical and in situ hybridization study in a mouse model. Neurol. Res. 36, 239–246. doi: 10.1179/1743132813Y.0000000289, PMID: 24512017

[ref141] Shin S. S. Bray E. R. Zhang C. Q. Dixon C. E. (2011). Traumatic brain injury reduces striatal tyrosine hydroxylase activity and potassium-evoked dopamine release in rats. Brain Res. 1369, 208–215. doi: 10.1016/j.brainres.2010.10.096, PMID: 21047500 PMC3014391

[ref142] Shuanglong Z. Jiangyuan Y. Meng N. Zheng W. Yunshui Z. Wei S.. (2024). A meta-analysis of cognitive and functional outcomes in severe brain trauma cases. Front. Behav. Neurosci. 18:1349672. doi: 10.3389/fnbeh.2024.1349672, PMID: 38549619 PMC10972858

[ref143] Singh A. Prajapati H. P. Kumar R. Singh N. P. Kumar A. (2022). Prognostic role of catecholamine in moderate-to-severe traumatic brain injury: a prospective observational cohort study. Asian J. Neurosur. 17, 435–441. doi: 10.1055/s-0042-1757217, PMID: 36398173 PMC9665989

[ref144] Slowinski A. Coetzer R. Byrne C. (2019). Pharmacotherapy effectiveness in treating depression after traumatic brain injury: a meta-analysis. J. Neuropsychiatr. Clin. Neurosci. 31, 220–227. doi: 10.1176/appi.neuropsych.18070158 30636565

[ref145] Small K. M. Nunes E. Hughley S. Addy N. A. (2016). Ventral tegmental area muscarinic receptors modulate depression and anxiety-related behaviors in rats. Neurosci. Lett. 616, 80–85. doi: 10.1016/j.neulet.2016.01.057, PMID: 26828299 PMC4798862

[ref146] Sönmez Ü. Sönmez A. Erbil G. Tekmen I. Baykara B. (2007). Neuroprotective effects of resveratrol against traumatic brain injury in immature rats. Neurosci. Lett. 420, 133–137. doi: 10.1016/j.neulet.2007.04.070, PMID: 17531385

[ref147] Spijker J. Claes S. (2014). Stemmingsstoornissen in de DSM-5 [mood disorders in the DSM-5]. Tijdschr. Psychiatr. 56, 173–176, PMID: 24643826

[ref148] Stein M. B. Jain S. Giacino J. T. Levin H. Dikmen S. Nelson L. D.. (2019). Risk of posttraumatic stress disorder and major depression in civilian patients after mild traumatic brain injury: a TRACK-TBI study. JAMA Psychiatry 76, 249–258. doi: 10.1001/jamapsychiatry.2018.4288, PMID: 30698636 PMC6439818

[ref149] Tan L. Ge G. Tang J. Fu C. Duanmu W. Chen Y.. (2015). Amantadine preserves dopamine levels and attenuates depression-like behavior induced by traumatic brain injury in rats. Behav. Brain Res. 279, 274–282. doi: 10.1016/j.bbr.2014.10.037, PMID: 25447294

[ref150] Tanaka H. Ehara A. Nakadate K. Yoshimoto K. Shimoda K. Ueda S. (2019). Behavioral, hormonal, and neurochemical outcomes of neonatal repeated shaking brain injury in male adult rats. Physiol. Behav. 199, 118–126. doi: 10.1016/j.physbeh.2018.11.025, PMID: 30465805

[ref151] Tanaka K. Ogawa N. Asanuma M. Kondo Y. (1997). Thyrotropin releasing hormone prevents abnormalities of cortical acetylcholine and monoamines in mice following head injury. Regul. Pept. 70, 173–178. doi: 10.1016/s0167-0115(97)01013-6 9272630

[ref153] Traeger J. Hoffman B. Misencik J. Hoffer A. Makii J. (2020). Pharmacologic treatment of neurobehavioral sequelae following traumatic brain injury. Crit. Care Nurs. Q. 43, 172–190. doi: 10.1097/CNQ.0000000000000301, PMID: 32084061

[ref154] Tsuda S. Golam M. Hou J. Nelson R. Bernavil P. Richardson K.. (2020). Altered monoaminergic levels, spasticity, and balance disability following repetitive blast-induced traumatic brain injury in rats. Brain Res. 1747:147060. doi: 10.1016/j.brainres.2020.147060, PMID: 32828734 PMC10424094

[ref155] Turner B. M. Paradiso S. Marvel C. L. Pierson R. Boles Ponto L. L. Hichwa R. D.. (2007). The cerebellum and emotional experience. Neuropsychologia 45, 1331–1341. doi: 10.1016/j.neuropsychologia.2006.09.023 17123557 PMC1868674

[ref156] Tye K. M. Mirzabekov J. J. Warden M. R. Ferenczi E. A. Tsai H. C. Finkelstein J.. (2013). Dopamine neurons modulate neural encoding and expression of depression-related behavior. Nature 493, 537–541. doi: 10.1038/nature11740, PMID: 23235822 PMC4160519

[ref157] Uiterwijk D. Stargatt R. Humphrey S. Crowe S. F. (2022). The relationship between cognitive functioning and symptoms of depression, anxiety, and post-traumatic stress disorder in adults with a traumatic brain injury: a meta-analysis. Neuropsychol. Rev. 32, 758–806. doi: 10.1007/s11065-021-09524-1, PMID: 34694543

[ref158] Vadlamani A. Albrecht J. S. (2020). Severity of traumatic brain injury in older adults and risk of ischemic stroke and depression. J. Head Trauma Rehabil. 35, E436–E440. doi: 10.1097/HTR.0000000000000561, PMID: 32108711 PMC7891874

[ref159] Van Bregt D. R. Thomas T. C. Hinzman J. M. Cao T. Liu M. Bing G.. (2012). Substantia nigra vulnerability after a single moderate diffuse brain injury in the rat. Exp. Neurol. 234, 8–19. doi: 10.1016/j.expneurol.2011.12.003, PMID: 22178300 PMC3294202

[ref160] Vecht C. J. van Woerkom C. A. Teelken A. W. Minderhoud J. M. (1975). Homovanillic acid and 5-hydroxyindoleacetic acid cerebrospinal fluid levels. A study with and without probenecid administration of their relationship to the state of consciousness after head injury. Arch. Neurol. 32, 792–797. doi: 10.1001/archneur.1975.00490540036004 1203031

[ref161] Verduzco-Mendoza A. Carrillo-Mora P. Avila-Luna A. Gálvez-Rosas A. Olmos-Hernández A. Mota-Rojas D.. (2021). Role of the dopaminergic system in the striatum and its association with functional recovery or rehabilitation after brain injury. Front. Neurosci. 15:693404. doi: 10.3389/fnins.2021.693404, PMID: 34248494 PMC8264205

[ref162] Vonder Haar C. Ferland J. M. N. Kaur S. Riparip L. K. Rosi S. Winstanley C. A. (2019a). Cocaine self-administration is increased after frontal traumatic brain injury and associated with neuroinflammation. Eur. J. Neurosci. 50, 2134–2145. doi: 10.1111/ejn.14123, PMID: 30118561 PMC6517083

[ref163] Vonder Haar C. Martens K. M. Bashir A. McInnes K. A. Cheng W. H. Cheung H.. (2019b). Repetitive closed-head impact model of engineered rotational acceleration (CHIMERA) injury in rats increases impulsivity, decreases dopaminergic innervation in the olfactory tubercle and generates white matter inflammation, tau phosphorylation and degeneration. Exp. Neurol. 317, 87–99. doi: 10.1016/j.expneurol.2019.02.012 30822421

[ref164] Wagner A. K. Chen X. Kline A. E. Li Y. Zafonte R. D. Dixon C. E. (2005b). Gender and environmental enrichment impact dopamine transporter expression after experimental traumatic brain injury. Exp. Neurol. 195, 475–483. doi: 10.1016/j.expneurol.2005.06.009, PMID: 16023635

[ref165] Wagner A. K. Drewencki L. L. Chen X. Santos F. R. Khan A. S. Harun R.. (2009). Chronic methylphenidate treatment enhances striatal dopamine neurotransmission after experimental traumatic brain injury. J. Neurochem. 108, 986–997. doi: 10.1111/j.1471-4159.2008.05840.x, PMID: 19077052 PMC2692956

[ref166] Wagner A. K. Postal B. A. Darrah S. D. Chen X. Khan A. S. (2007b). Deficits in novelty exploration after controlled cortical impact. J. Neurotrauma 24, 1308–1320. doi: 10.1089/neu.2007.0274, PMID: 17711392

[ref167] Wagner A. K. Ren D. Conley Y. P. Ma X. Kerr M. E. Zafonte R. D.. (2007a). Sex and genetic associations with cerebrospinal fluid dopamine and metabolite production after severe traumatic brain injury. J. Neurosurg. 106, 538–547. doi: 10.3171/jns.2007.106.4.538, PMID: 17432702

[ref168] Wagner A. K. Scanlon J. M. Becker C. R. Ritter A. C. Niyonkuru C. Dixon C. E.. (2014). The influence of genetic variants on striatal dopamine transporter and D2 receptor binding after TBI. J. Cereb. Blood Flow Metab. 34, 1328–1339. doi: 10.1038/jcbfm.2014.87, PMID: 24849661 PMC4126093

[ref169] Wagner A. K. Sokoloski J. E. Ren D. Chen X. Khan A. S. Zafonte R. D.. (2005a). Controlled cortical impact injury affects dopaminergic transmission in the rat striatum. J. Neurochem. 95, 457–465. doi: 10.1111/j.1471-4159.2005.03382.x 16190869

[ref170] Wen X. Wu X. Liu J. Li K. Yao L. (2013). Abnormal baseline brain activity in non-depressed Parkinson’s disease and depressed Parkinson’s disease: a resting-state functional magnetic resonance imaging study. PLoS One 8:e63691. doi: 10.1371/journal.pone.0063691, PMID: 23717467 PMC3661727

[ref171] Whelan-Goodinson R. Ponsford J. Johnston L. Grant F. (2009). Psychiatric disorders following traumatic brain injury: their nature and frequency. J. Head Trauma Rehabil. 24, 324–332. doi: 10.1097/HTR.0b013e3181a712aa 19858966

[ref173] Wilson M. S. Chen X. Ma X. Ren D. Wagner A. K. Reynolds I. J.. (2005). Synaptosomal dopamine uptake in rat striatum following controlled cortical impact. J. Neurosci. Res. 80, 85–91. doi: 10.1002/jnr.20419, PMID: 15704194

[ref174] Womack K. B. Dubiel R. Callender L. Dunklin C. Dahdah M. Harris T. S.. (2020). 123 I-Iofluopane single-photon emission computed tomography as an imaging biomarker of pre-synaptic dopaminergic system after moderate-to-severe traumatic brain injury. J. Neurotrauma 37, 2113–2119. doi: 10.1089/neu.2019.6892 32216525

[ref175] Woolf P. D. Hamill R. W. Lee L. A. Cox C. McDonald J. V. (1987). The predictive value of catecholamines in assessing outcome in traumatic brain injury. J. Neurosurg. 66, 875–882. doi: 10.3171/jns.1987.66.6.0875, PMID: 3572517

[ref176] Woolf P. D. Hamill R. W. Lee L. A. McDonald J. V. (1988). Free and total catecholamines in critical illness. Am. J. Phys. 254, E287–E291. doi: 10.1152/ajpendo.1988.254.3.E287, PMID: 3348389

[ref177] Writer B. W. Schillerstrom J. E. (2009). Psychopharmacological treatment for cognitive impairment in survivors of traumatic brain injury: a critical review. J. Neuropsychiatr. Clin. Neurosci. 21, 362–370. doi: 10.1176/jnp.2009.21.4.362, PMID: 19996244

[ref178] Xu X. Cao S. Chao H. Liu Y. Ji J. (2016). Sex-related differences in striatal dopaminergic system after traumatic brain injury. Brain Res. Bull. 124, 214–221. doi: 10.1016/j.brainresbull.2016.05.010, PMID: 27210290

[ref179] Yan H. Q. Kline A. E. Ma X. Hooghe-Peters E. L. Marion D. W. Dixon C. E. (2001). Tyrosine hydroxylase, but not dopamine beta-hydroxylase, is increased in rat frontal cortex after traumatic brain injury. Neuroreport 12, 2323–2327. doi: 10.1097/00001756-200108080-00009 11496104

[ref180] Yan H. Q. Kline A. E. Ma X. Li Y. Dixon C. E. (2002). Traumatic brain injury reduces dopamine transporter protein expression in the rat frontal cortex. Neuroreport 13, 1899–1901. doi: 10.1097/00001756-200210280-00013, PMID: 12395087

[ref181] Yan H. Q. Ma X. Chen X. Li Y. Shao L. Dixon C. E. (2007). Delayed increase of tyrosine hydroxylase expression in rat nigrostriatal system after traumatic brain injury. Brain Res. 1134, 171–179. doi: 10.1016/j.brainres.2006.11.087, PMID: 17196177 PMC4017583

[ref182] Yang S. Y. Zhang S. Wang M. L. (1995). Clinical significance of admission hyperglycemia and factors related to it in patients with acute severe head injury. Surg. Neurol. 44, 373–377. doi: 10.1016/0090-3019(96)80243-6, PMID: 8553258

[ref183] Yehene E. Lichtenstern G. Harel Y. Druckman E. Sacher Y. (2020). Self-efficacy and acceptance of disability following mild traumatic brain injury: a pilot study. Appl. Neuropsychol. Adult 27, 468–477. doi: 10.1080/23279095.2019.1569523, PMID: 30806085

